# Dynamics of resistant genes and their relationship to resistance to wheat yellow mosaic disease in Japanese wheat germplasms

**DOI:** 10.1270/jsbbs.25058

**Published:** 2026-04-21

**Authors:** Fuminori Kobayashi, Shuhei Okada, Hisayo Kojima, Kenji Kawaguchi, Hidekazu Maejima, Masaya Fujita, Zenta Nishio

**Affiliations:** 1 Institute of Crop Science, National Agriculture and Food Research Organization, 2-1-2 Kannondai, Tsukuba, Ibaraki 305-8518, Japan; 2 Department of Agriculture, Tokyo University of Agriculture, Atsugi, Kanagawa 243-0034, Japan; 3 Research Faculty of Agriculture, Hokkaido University, Sapporo, Hokkaido 060-8589, Japan; 4 Hokkaido Agricultural Research Center, National Agriculture and Food Research Organization, 9-4 Shinseiminami, Memuro, Kasai, Hokkaido 082-0081, Japan; 5 Kyushu Okinawa Agricultural Research Center, National Agriculture and Food Research Organization, Izumi 496, Chikugo, Fukuoka 833-0041, Japan; 6 Nagano Prefecture Agricultural Experiment Station, 610 Yaemori, Suzaka, Nagano 382-0051, Japan; 7 Headquarters, National Agriculture and Food Research Organization, Tsukuba, Ibaraki 305-8517, Japan

**Keywords:** *Triticum aestivum* L., wheat yellow mosaic virus, infection-resistant genes, Japanese wheat core collection, resistance breeding

## Abstract

Wheat yellow mosaic (WYM) disease, caused by the wheat yellow mosaic virus (WYMV), significantly affects wheat production. Three WYMV pathotypes—I, II, and III—have been identified in Japan, each with a distinct geographical distribution and pathogenicity. To investigate the historical and geographical distribution of infection-resistant wheat varieties in Japan and the relevant genetics, we comprehensively evaluated the response to WYMV pathotypes and the genotypes conferring resistance in accessions of the Japanese wheat core collection. In the experimental fields harboring WYMV, most of the varieties that showed stable resistance were breeders lines; only a few were traditional varieties. Resistant accessions are particularly prevalent in northern Japan. These results suggest that resistant varieties have been developed through modern breeding, in which crossbreeding with foreign varieties has played a major role. Resistance-conferring genes on the chromosome 2DL and 5AL appeared to have been introduced during this breeding process, and the 5AL locus became widely distributed in Japanese varieties. Through genome-wide association studies, we identified a novel resistance-conferring locus on chromosome 7AS. Furthermore, the resistant haplotype on 7AS confers robust resistance to pathotype I when combined with the haplotype carried on 5AL. The results of this study contribute to the understanding of resistance-associated genetics in the context of WYM disease, highlighting the potential of particular genomes to confer robust and durable resistance in breeding efforts.

## Introduction

Wheat yellow mosaic (WYM) disease is a serious soil-borne disease affecting wheat production. It is caused by the wheat yellow mosaic virus (WYMV), which is transmitted by the plasmodiophorid *Polymyxa graminis*. WYMV causes characteristic yellow stripes on leaves and dwarfing, both of which significantly reduce wheat yield. The symptoms of WYMV are similar to those of the wheat spindle streak mosaic virus (WSSMV). The two viruses, both belonging to the genus *Bymovirus* (family Potyviridae), were differentiated based on their genome sequences (Lu *et al.* 1998, Namba *et al.* 1998); their genomes are composed of two types of single-strand RNAs, RNA1 and RNA2. WSSMV is widely distributed, mainly in North America and Europe, whereas WYMV is found only in Japan and China (Han *et al.* 2000, Inouye 1969).

In Japan, three WYMV pathotypes have been identified: I, II, and III. These pathotypes are classified based on their infectivity in standard varieties, and the differences are attributed to the type and combination of RNA1 and RNA2. Geographical distribution is also distinctive among the pathotypes; pathotype I is distributed in central and southwestern Japan, pathotype II is found in the northern Japan, including Tohoku and Hokkaido regions, and pathotype III is prevalent in Fukuoka, a part of Kyushu region (Kojima *et al.* 2019, Ohki *et al.* 2014, Ohto *et al.* 2006). Pathotype III is considered a mutant of pathotype I and its pathogenicity is stronger than that of pathotype I (Kojima *et al.* 2019, Ohki *et al.* 2014). To date, quantitative trait loci (QTLs) conferring resistance to WYMV have been identified on the chromosome arms 2AS, 2AL, 2DL, 3BS, 4DS, 5AL, 6DS, and 7BS (Kawaguchi *et al.* 2024, Kojima *et al.* 2015, Liu *et al.* 2005a, 2005b, Nishio *et al.* 2010, Suzuki *et al.* 2015, Xiao *et al.* 2016, Yamashita *et al.* 2020, Zhu *et al.* 2012). Of these, QTLs on 2DL, 3BS, and 5AL were considered to contribute to resistance to WYMV in Japan, and their effects on the three WYMV pathotypes were evaluated using 165 modern Japanese varieties released by the Ministry of Agriculture, Forestry, and Fisheries (Kojima *et al.* 2019). The following resistance models have been considered: 1) the resistance associated with wheat chromosome arm (locus) 2DL is completely effective against pathotypes I and III but only partially effective against pathotype II, and 2) the loci 3BS and 5AL are associated with partial resistance to pathotypes II and I, respectively. The relationship between resistance genes and degree of WYMV infection is useful in planning resistance breeding of wheat varieties. Recently, additional QTLs associated with resistance to Japanese WYMV pathotypes were identified on chromosome arms 2AS and 6DS: *Q.Ymhk* for pathotypes II and III, and *Qym4* for pathotype II (Kawaguchi *et al.* 2024, Yamashita *et al.* 2020). The resistance conferred by these QTLs was found to be effective against each of the viral pathotype and is expected to be utilized in wheat resistance breeding.

It has long been known that the severity of WYM disease is greatly influenced by environmental factors (Saito and Okamoto 1964). In particular, temperature is recognized as a key factor in this disease, and its effect on WYMV ecology has been investigated in detail (Ohto 2005). The favorable temperature for virus propagation in wheat plants is approximately 10°C, and temperatures in the range of 5°–10°C are favorable for disease progression. WYM disease is sensitive to temperature conditions; therefore, temperature fluctuations (due to climate change) from autumn to winter, when viral infection, propagation, and movement occur in wheat, have a significant impact on the incidence of WYM disease. Furthermore, there is a possibility of the emergence of new pathotypes and the breakdown of existing resistance in wheat varieties.

Understanding the diversity of genetic factors responsible for WYMV resistance is essential in order to prepare for changes in infection risk brought about by changing climatic conditions. In previous studies of WYMV-resistant genes, the focus has been on commercial wheat varieties, whose genetic diversity has been reduced through modern breeding processes, leaving little opportunity of discovering additional resistance-associated genes (Fu 2015, Lopes *et al.* 2015, Louwaars 2018, Tanksley and McCouch 1997). However, landraces and old varieties often have high genetic diversity compared to current commercial varieties and are likely to carry unknown useful genes (Newton *et al.* 2010, Wingen *et al.* 2017). These germplasm are potentially useful breeding materials; however, their resistance to WYMV pathotypes is unknown. Japanese wheat is adapted to the unique environmental conditions of the eastern region of the Eurasian continent, such as high temperature and humidity, which are different from those of West Asia, where wheat originated (Oda 2015). In addition, Japanese wheat has genetically different characteristics from those of the other major wheat-growing regions, namely Europe, North and South America, and Australia (Shimizu *et al.* 2021, Walkowiak *et al.* 2020). Consequently, indigenous Japanese germplasm has great potential as a genetic resource for wheat improvement.

In this study, we comprehensively evaluated the infectivity and distribution of wheat genes conveying resistance to WYMV pathotypes using the Japanese wheat core collection (JWC) of the National Agriculture and Food Research Organization (NARO) (Kojima *et al.* 2017). JWC contains 96 accessions, including landraces and old varieties, selected based on the history of wheat cultivation and breeding from the early 1900s to the 1990s in Japan. We identified novel genes associated with WYMV resistance in Japanese wheat. The relationships between the distribution of resistance genes in the JWC accessions and the three WYMV pathotypes suggested a historical process of resistance gene accumulation and its impact on WYM disease. This overview of the dynamics of resistance genes in past breeding has enabled us to propose one aspect of genetic improvement methods for the future development of robust virus-resistant wheat varieties.

## Materials and Methods

### Plant materials and field experiments for wheat yellow mosaic resistance

The Japanese wheat core collection (JWC) provided by the NARO genebank were used in this study. JWC is representative of Japanese wheat genetic resources, consisting of 75 breeders lines, 11 landraces and 9 unknown lines, as well as the cv. ‘Chinese Spring’ (CS) (Kobayashi *et al.* 2016, Kojima *et al.* 2017, Mizuno *et al.* 2024).

Field trials for WYMV infectivity were conducted during two growing seasons: 2016–2017 and 2017–2018. In each season, JWC accessions were planted in a pathotype I-harboring field in the Utsunomiya, Tochigi, Kanto region in mid-October, and in a pathotype III-harboring field in the Yanagawa, Fukuoka, Kyushu region in mid-November. Each experimental unit consisted of a single 50-cm-long row with 5 cm between each plant. Ten individuals were grown per replicate and sampling was performed in two replicates. The field trial was also conducted in a pathotype II-harboring field in the Obihiro, Hokkaido region during the 2022–2023 season. Ten seeds were sown on September 28, 2022, in a plot consisting of 50-cm-long rows and 27-cm row spacing. Additional examinations of several accessions, JWC23, JWC41, JWC48, JWC54, JWC66, JWC79, JWC85, JWC96 and ‘Kitahonami’, were conducted in nursery fields contaminated with pathotype I in the Matsumoto, Nagano, Chubu region during the 2020–2021, 2021–2022, 2022–2023 and 2023–2024 seasons. Ten seeds were sown per plot in late September, with 50 cm between rows. Sampling was performed with two replicates. The experimental fields used in this study were identical to those used in previous studies (Kawaguchi *et al.* 2024, Kojima *et al.* 2019, Ohki *et al.* 2014).

### WYMV detection by ELISA

As disease symptoms characterized by a yellow–striped mosaic pattern are often difficult to distinguish from leaf yellowing caused by cold and frost stress, a double-antibody sandwich (DAS)-ELISA with polyclonal antisera against WYMV was used to confirm WYMV infection. Leaf samples were collected in mid-March of each growing season at Utsunomiya and Matsumoto, and in late February in Yanagawa. In Obihiro, leaf sampling was carried out three times: December 12, 2022, April 7, 2023, and April 21, 2023. ELISA was conducted using the procedure described by Kojima *et al.* (2019). For each of the 96 accessions, crude extracts were prepared for ELISA analysis from bulked leaf samples, each consisting of five leaves collected from a single plot, by grinding in phosphate-buffered saline containing Tween (PBST). The absorbance was measured at 405 nm. Samples with more than twice the absorbance of negative control (non-infected) leaves were considered WYMV-infected. For each of the two pathotypes, I and III, the accessions that showed resistance during the two growing seasons were evaluated as having stable resistance. Regarding pathotype II, samples that had more than twice the absorbance of negative control leaves were assessed as WYMV-infected, whereas accessions that showed no infection in three samplings were evaluated as resistant. Data from the Obihiro nursery field were used as supplemental data in this study because they were obtained for only one season.

### DNA extraction and polymorphism analysis of molecular markers

Genomic DNA was extracted from leaves using a DNeasy Plant Mini Kit (Qiagen, Germany). To determine the haplotypes of the QTLs conferring WYMV resistance in the 96 accessions, genotyping with flanking markers of each QTL was conducted. The target QTLs *Q.Ymhk*, *Q.Ymym*, *Qym2*, *QYm.njau-5A.1*, and *Qym4*, were located on chromosome arms 2AS, 2DL, 3BS, 5AL, and 6DS, and their flanking markers, *snp4212*, *SPT20446*, *wmc754*, *wmc415*, and *cfd49*, were used for the genotyping, respectively (Kawaguchi *et al.* 2024, Kobayashi *et al.* 2020a, Somers *et al.* 2004, Suzuki *et al.* 2015, Yamashita *et al.* 2020, Zhu *et al.* 2012). The marker information, including primer sequences, is provided in Supplemental Table 1.

Genotyping of the *snp4212* marker for the 2AS locus was performed using the Kompetitive allele-specific PCR (KASP) assay as described by Mizuno *et al.* (2021) and Kawaguchi *et al.* (2024). PCR was performed in a 6-μL reaction volume containing 2.5 μL KASP master mix (LGC Biosearch Technologies, UK), 0.07 μL KASP primer mix (12 μM FAM primer, 12 μM HEX primer, and 30 μM common primer), 1 μL DNA template with a concentration of 20 ng/μL, and 2.43 μL deionized water. Cycling conditions for the marker were: 94°C for 15 min, followed by 10 cycles at 94°C for 20 s and 61°C for 1 min, followed by 38 cycles at 94°C for 20 s and 55°C for 1 min. Fluorescence endpoints were read on a CFX384 (Bio-Rad, Hercules, CA, USA), and genotypes were determined using the allele discrimination mode in the CFX Manager v.3.1 software (Bio-Rad).

Genotyping of other markers was performed using PCR. Accessions having the same specific bands as ‘Yumechikara’, ‘Madsen’, ‘Nishikaze komugi’ and ‘OW104’ for *SPT20446*, *wmc754*, *wmc415*, and *cfd49* markers were assumed to carry the resistance haplotype on chromosome loci 2DL, 3BS, 5AL and 6DS, respectively. PCR was conducted using a T100 thermal cycler (Bio-Rad) and GoTaq DNA polymerase (Promega Corp., Madison, WI, USA). The PCR conditions were as follows: denaturation at 95°C for 1 min, followed by 35 cycles of the denaturation at 95°C for 30 s, annealing for 30 s, extension at 72°C for 30 s, and then final extension at 72°C for 5 min. The annealing temperatures for each marker are listed in Supplemental Table 1. The amplicons of *SPT20446* marker were separated on a 2.0% agarose gel and visualized using SYBR Safe DNA Gel Stain (Invitrogen, Carlsbad, CA, USA). The *wmc754*, *wmc415* and *cfd49* markers were separated using a capillary electrophoresis system (LabChip GX Touch HT, PerkinElmer, Inc., Waltham, MA, USA) with a DNA5K/RNA/CZE chip (PerkinElmer).

### Genome-wide association study (GWAS) for WYMV resistance

Genome-wide SNP data for the JWC accessions were previously generated using the genotyping-by-sequencing method (Kobayashi *et al.* 2016). The amplicon-seq and ddRAD-seq methods have been used for additional SNP calling (Mizuno *et al.* 2024). Consequently, 30,217 SNPs assigned along the reference genome sequence of the CS, IWGSC RefSeq v2.1 (Zhu *et al.* 2021), have been generated (Mizuno *et al.* 2024). Phenotype data were obtained from the ELISA results for each year for pathotype I and pathotype III resistance. For the analysis, the ELISA results (“+” and “–”) shown in Supplemental Table 2 were converted to numerical scores: “0” for susceptible and “1” for resistant (Hourcade *et al.* 2019). Genotype data were loaded into TASSEL 5 (Bradbury *et al.* 2007) and filtered with a minimum allele frequency (maf) ≥ 0.05, resulting in 27,781 SNPs for analysis. These SNPs were loaded into R software using the vcfR package (Knaus and Grünwald 2017), and a GWAS was performed up to the fourth principal component (Stacklies *et al.* 2007) using the GWA function of rrBLUP (Endelman 2011). The significance level was set at *p* < 0.001 (–log10 [0.001]). Linkage disequilibrium between markers was evaluated by calculating the correlation coefficient (r) between markers, and those with |r| ≥ 0.5 were in linkage disequilibrium relationship.

## Results

### WYMV infection in JWC accessions

To evaluate the response of 96 JWC accessions against the two WYMV pathotypes, I and III, we performed ELISA using leaves collected from each of the two experimental fields in Utsunomiya and Yanagawa during the two growing seasons. The results showed that 40 and 37 accessions were resistant to pathotype I, and 19 and 12 accessions were resistant to pathotype III during the 2016–2017 and 2017–2018 seasons, respectively (Table 1). Pathotype I WYMV infection was confirmed in 56 and 59 accessions, and 77 and 84 accessions harbored pathotype III infections during the 2016–2017 and 2017–2018 seasons, respectively (Table 1). Pathotypes I and III had higher infection rates in 2017–2018 than in 2016–2017 (Table 1). Subsequently, WYMV infection of pathotype II was also evaluated by ELISA in the 2022–2023 season. Of the three tests during this season, 39 accessions did not show any infection, whereas 37 accessions showed clear infection (Supplemental Table 2).

Eleven accessions, ‘Koshigun zairaishu’ (JWC23), ‘Aso zairai’ (Yuubou Kappu) (JWC41), ‘Honkei 275’ (JWC47), ‘Hokkai 240’ (JWC48), ‘Igachikugo Oregon’ (JWC52), ‘Norin 10’ (JWC57), ‘Norin 16’ (JWC58), ‘Norin 27’ (JWC60), ‘Takune komugi’ (JWC72), ‘Furutsumasari’ (JWC76), and ‘Hachiman komugi’ (JWC95), were resistant to each of the two pathotypes during both growing seasons, of which six accessions, JWC41, JWC47, JWC52, JWC60, JWC76, and JWC95, were potentially resistant to pathotype II (Table 2, Supplemental Table 2). Twenty-two accessions were stably resistant to pathotype I but susceptible to pathotype III. ‘Norin 1’ (JWC54), which exhibited resistance to pathotype III, was infected with pathotype I in Utsunomiya in 2016–2017 season but showed stable resistance to another pathotype I in Matsumoto over four growing seasons (Supplemental Table 3). Therefore, among the JWC accessions that were susceptible to pathotype I, none showed stable resistance to pathotype III. There were 62 accessions that were susceptible to both pathotypes I and III, whereas 29 were not infected with pathotype II (Table 2).

### Geographical and historical distribution of resistant varieties

**Fig. 1.**
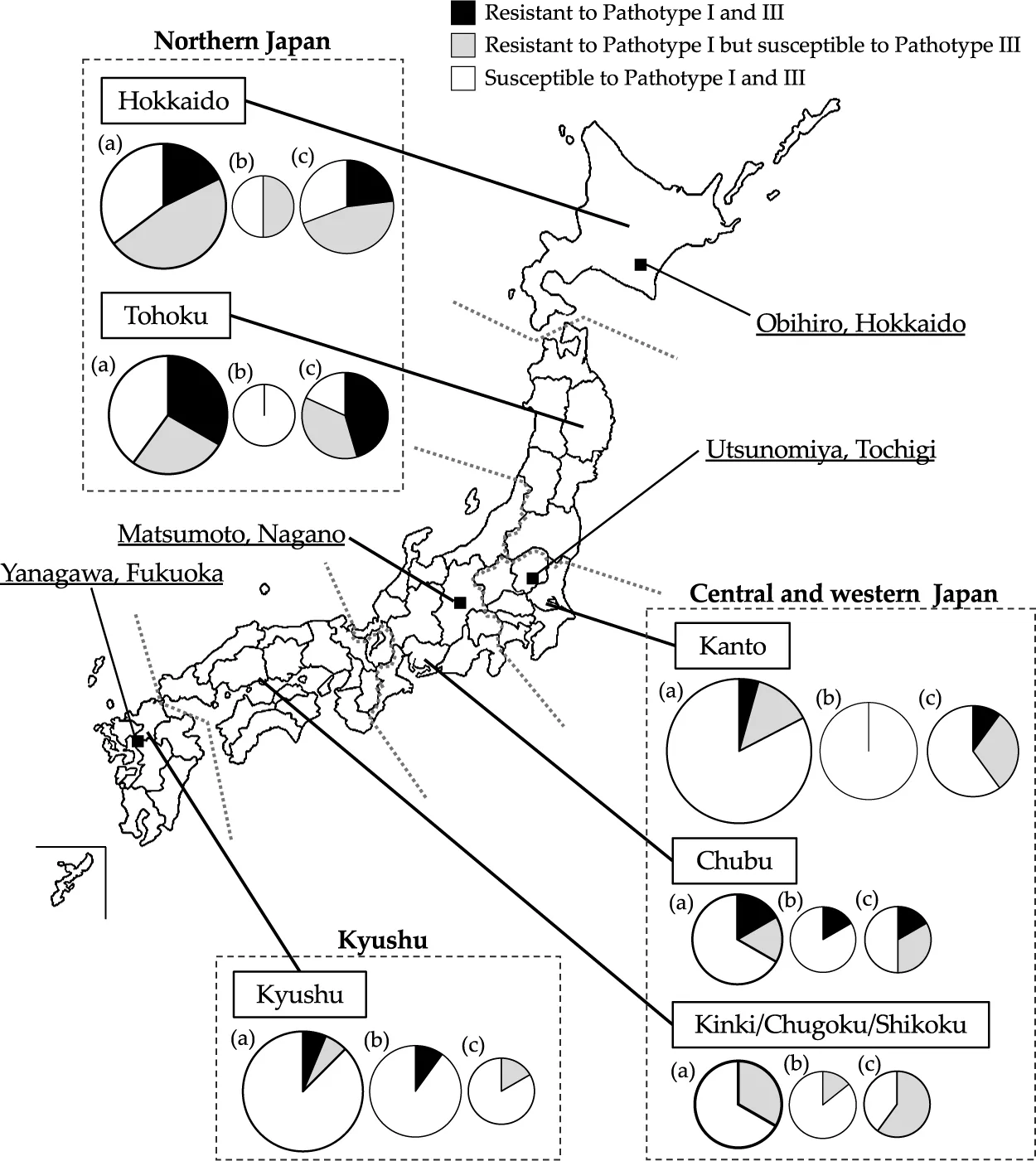
Geographical distribution of resistant varieties of 95 accessions of the Japanese wheat core collection (JWC), excluding cv. ‘Chinese Spring’. The size of the pie chart corresponds to the number of accessions: (a) Total accession in each region, (b) landraces and pure-selected lines, (c) breeders lines. Place names of experimental fields are underlined. The dotted lines indicate boundaries of each region.

We investigated the geographical distribution of the JWC accessions that exhibited stable resistance to WYMV. In central and western Japan (Kanto, Chubu, Kinki, Chugoku, and Shikoku regions) in which pathotype I is distributed, 12 of the 47 accessions bred in this region showed resistance to pathotype I, among which JWC23, JWC52, and JWC58 were also resistant to pathotype III (Fig. 1, Table 3, Supplemental Table 2). There were 24 accessions that were not infected with pathotype II, including five accessions that were resistant to pathotype I (Supplemental Tables 2, 4). Of the 32 accessions bred in northern Japan (Hokkaido and Tohoku regions) in which pathotype II is distributed, 20 accessions were resistant to pathotype I, including eight accessions exhibiting resistance to pathotype III: JWC47, JWC48, JWC54, JWC57, JWC60, JWC72, JWC76, and JWC95 (Fig. 1, Table 3, Supplemental Table 2). Only seven accessions were not infected with pathotype II, including some of the accessions mentioned above: JWC47, JWC60, JWC76, and JWC95 (Supplemental Tables 2, 4). Although pathotype III is distributed in part of the Kyushu region, we focused on 16 accessions bred throughout the Kyushu region. Only two accessions, JWC41 and ‘Abukumawase’ (JWC81), showed resistance to pathotype I, and one of them, JWC41, was also resistant to pathotypes III (Fig. 1, Table 3, Supplemental Table 2). WYMV pathotype II did not infect 8 of the 16 accessions, including JWC41 (Supplemental Tables 2, 4).

According to previous reports by Kobayashi *et al.* (2016) and Kojima *et al.* (2017), the 95 JWC accessions, excluding CS (JWC96), were classified into two categories based on breeding methods: landraces and pure selected lines (44 accessions) and breeders lines (51 accessions) (Supplemental Table 2). Landraces and pure selected lines were widely cultivated in various parts of Japan, mainly from the 1900s to the 1930s, or even before that. Five out of 44 accessions are resistant to pathotype I, in which JWC23 and JWC41 with resistance to pathotype III were included (Fig. 1, Table 3, Supplemental Table 2). These two accessions also showed resistance in Matsumoto (Supplemental Table 3). Breeders lines have been bred through crossbreeding since the 1910s, and 29 accessions were resistant to pathotype I, including 10 accessions resistant to pathotype III (Fig. 1, Table 3). These results show that there was a large difference in the historical distribution of resistant varieties between the two categories (11.4% and 56.9% for pathotype I and 4.5% and 19.6% for pathotype III), with many resistant varieties appearing in the late 1930s and from the 1950s to the 1970s (Table 3, Supplemental Table 2). In the breeders lines, the geographical distribution of accessions resistant to pathotype I was the highest in northern Japan (75.0%), followed by central and western Japan (47.6%), and Kyushu (16.7%) (Table 3). This trend was similar for the accessions resistant to pathotype III (33.3%, 9.5%, and 0%, respectively; Table 3). In contrast, 20 and 19 accessions in the two categories were not infected with pathotype II, the frequency of which was higher in the landraces and pure selected lines (45.5%) than in breeders lines (37.3%) (Supplemental Table 4).

### Distribution of resistant genes in JWC accessions

**Fig. 2.**
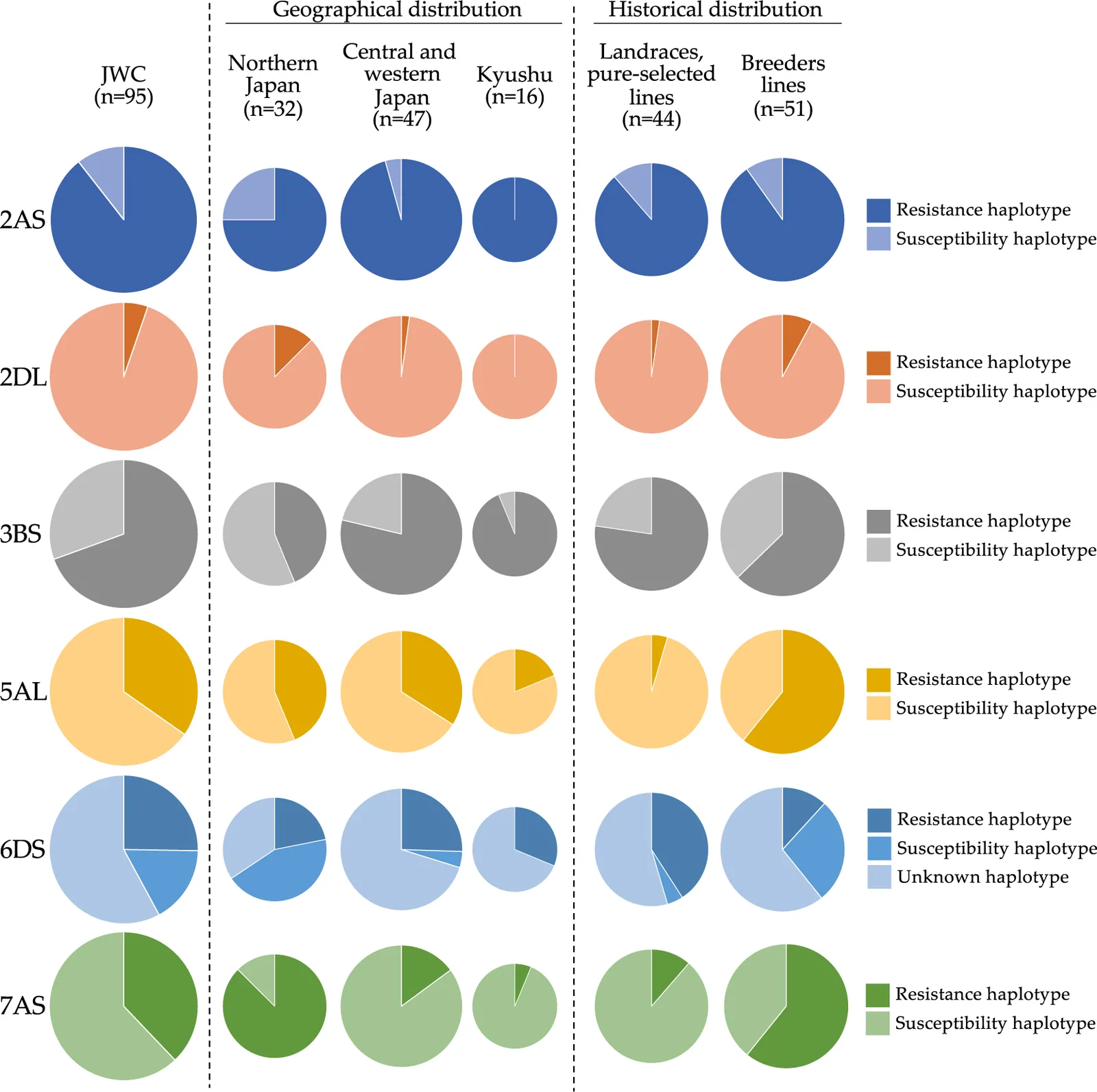
Distribution of resistant genes in JWC accessions, excluding cv. ‘Chinese Spring’. The size of the pie chart corresponds to the number of accessions.

To identify how JWC varieties acquired WYMV resistance, we genotyped 96 accessions using three PCR markers tightly linked to the resistance-associated QTLs, *Q.Ymym*, *Qym2* and *QYm.njau-5A.1*, on chromosome arms 2DL, 3BS, and 5AL, respectively (Kobayashi *et al.* 2020a, Suzuki *et al.* 2015, Zhu *et al.* 2012). In the 96 accessions, resistance haplotypes on 2DL, 3BS, and 5AL were detected in 5, 67, and 33 accessions, respectively (Fig. 2, Supplemental Table 5). The accessions with resistance haplotype on 2DL were ‘Dawson 1’ (JWC03), ‘Igachikugo Oregon’ (JWC52), ‘Norin 27’ (JWC60), ‘Furutsu masari’ (JWC76) and ‘Hachiman komugi’ (JWC95), all of which except JWC52 are cultivated in northern Japan. Additionally, JWC03 was classified as a pure selected line, whereas the other lines were breeders lines (Supplemental Table 2).

Of the 66 accessions with resistance haplotype on 3BS, 14, 37, and 15 were from northern Japan, central and western Japan, and the Kyushu region, respectively (Fig. 2, Supplemental Table 5). The distribution frequency of the 3BS haplotype was higher in central and western Japan (78.7%) and the Kyushu region (93.8%) than in northern Japan (43.8%). Historical distribution showed that 34 and 32 accessions were categorized into the landraces and pure selected lines, and the breeders lines, respectively (Supplemental Table 5). Therefore, the resistant haplotype on 3BS was mainly distributed from the Kanto to Kyushu regions, regardless of the breeding history.

The 33 accessions with resistance haplotype on 5AL were categorized as follows: 14 from northern Japan, 16 from central and western Japan, and 3 from the Kyushu region (Fig. 2, Supplemental Table 5). The frequency of the 5AL haplotype was highest in northern Japan (43.8%), followed by central and western Japan (34.0%) and the Kyushu region (18.8%). Its historical distribution was distinctive, with 31 of 33 accessions being breeders lines (Supplemental Table 5). These results indicate that the resistance haplotype on 5AL was distributed mainly from northern to western Japan during the breeding process.

Recently, two resistance QTLs, *Q.Ymhk* and *Qym4*, have been identified on chromosome arms 2AS and 6DS from winter wheat varieties ‘Hokkai 240’ and ‘OW104, ’ respectively (Kawaguchi *et al.* 2024, Yamashita *et al.* 2020). Therefore, we added flanking markers for the QTLs to the JWC accessions. Genotyping using the KASP marker *snp4212* for *Q.Ymhk* showed that 85 accessions and CS had the same haplotype on 2AS as ‘Hokkai 240’ (Fig. 2, Supplemental Table 5). Its frequency was the highest among all the loci tested in this study. Of the remaining 10 accessions with susceptibility haplotypes, 8 were varieties from northern Japan.

Genotyping with *cfd49* marker adjacent to *Qym4* revealed that 26 accessions had resistance haplotypes on 6DS, 14 had susceptibility haplotypes, and the rest were indistinguishable (Fig. 2, Supplemental Table 5). The 26 accessions with the resistance haplotype consisted of 7, 14, and 5 from northern Japan, central and western Japan, and the Kyushu region, respectively, with regional frequencies of 21.9%, 29.8%, and 31.3%, respectively. Its historical distribution was distinctive; 18 of 26 accessions were landraces and pure selected lines, and the remaining eight breeders lines were bred before the 1940s (Supplemental Tables 2, 5). These results indicate that the resistance haplotype on 6DS was mainly distributed in relatively old accessions, and its distribution frequency was low in northern Japan.

### Detection of marker–trait associations (MTAs) for WYMV resistance in JWC by GWAS

**Fig. 3.**
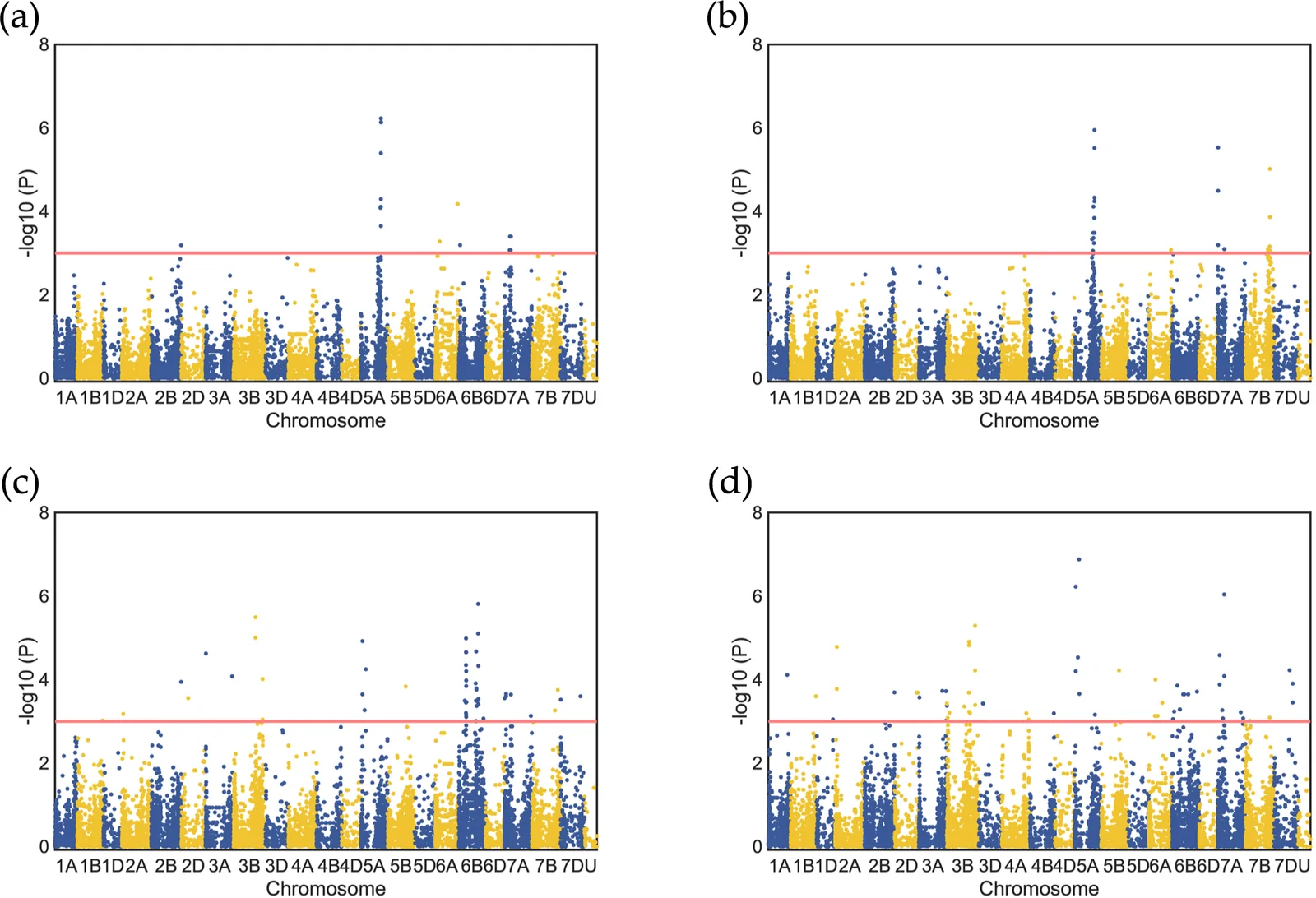
Genome-wide association analysis (GWAS) of resistance to wheat yellow mosaic virus (WYMV) in 96 JWC varieties. The x-axis represents the chromosomes in sequential order. The red line indicates the threshold (–log10(p) = 3): (a) Pathotype I resistance in 2016–2017 season; (b) Pathotype I resistance in 2017–2018 season; (c) Pathotype III resistance in 2016–2017 season; (d) Pathotype III resistance in 2017–2018 season.

To identify new gene loci responsible for WYMV resistance, a marker-trait association analysis was performed using a genome-wide SNP dataset (Mizuno *et al.* 2024). A total of 140 and 296 MTAs with *p* values ≤0.001 were detected from the ELISA data of two seasons for pathotype I and III, respectively (Fig. 3, Supplemental Tables 6, 7). Among these, there were 12 and 9 MTAs for traits over the significance level of 5% after Bonferroni multiple test correction (*p* = 1.655 × 10^–6^), respectively.

The 12 MTAs responsible for pathotype I resistance in 2016–2017 season were located on chromosome arm 5AL (537,415,352–539,737,733 bp), of which the marker *G_Chr5A_537415352* was significantly detected in two seasons and other MTAs were also detected in 2017–2018 season with *p* values ≤0.001 (Fig. 3a, 3b, Supplemental Table 6). Additionally, these markers showed a high correlation coefficient (|r| ≥ 0.9) with the genotype of *wmc415* (535,809,651–535,809,770 bp) linked to the *QYm.njau-5A.1* and were located very close to each other, indicating that these markers represent the resistance locus on 5AL (Supplemental Table 6). Of the significant nine MTAs responsible for pathotype III resistance, three, four, and two were located on chromosomes 5A, 6B, and 7A, respectively (Fig. 3c, 3d, Supplemental Table 7). The three MTAs on 5A were different from the MTAs detected in the response to pathotype I resistance, due to their chromosomal position and a correlation coefficient with the genotype of *wmc415* (|r| ≤ 0.2). Among the chromosomal regions with nine MTAs, other MTAs with *p* values ≤0.001 for pathotypes I and III resistance were detected in the two MTA-containing region on chromosome arm 7AS (approximately 172–178 Mb) over two seasons, but not on the 5A and 6B (Supplemental Tables 6, 7). This suggests the existence of a QTL for WYMV resistance in this region of chromosome arm 7AS.

For the most significant marker *G_Chr7A_173288218* in this region, SNP genotypes of ‘C’ (reference-type) or ‘T’ (alternative-type) of JWC revealed that accessions with genotype ‘T’ had lower WYMV infection rates for both pathotypes I and III (Table 4), therefore the alternative-type was defined as the resistance haplotype. The resistance haplotype was found in 36 accessions, of which 28 were varieties from northern Japan (Supplemental Table 5). Furthermore, its historical distribution showed that 31 out of 36 accessions were breeders lines, and the remaining five accessions were JWC01, JWC02, JWC03, JWC04 and JWC41; with the exception of JWC41, these are landraces in Hokkaido region (Supplemental Table 2). Thus, the resistance haplotype of *G_Chr7A_173288218* was mainly distributed in breeders lines in northern Japan.

### Relationships between resistance genotype and WYMV resistance

Three loci, 2DL, 3BS, and 5AL, have been evaluated for their effects on WYMV pathotypes (Kojima *et al.* 2019). As in a previous report, accessions with the resistance haplotype on 2DL tended to show stable resistance to pathotypes I and III over the two seasons (Table 5). Accessions with the resistance haplotype on 5AL were highly resistant to pathotype I (72.7%), but less resistant to pathotype III (12.1%) (Table 5). In contrast, most accessions with 3BS resistance haplotype were highly susceptible to pathotypes I and III (Table 5). Similarly, 13 of the 14 accessions with susceptibility haplotypes at the three loci were infected with both pathotypes I and III (Supplemental Table 8). These results are largely consistent with those of previous studies in which the relationship between genotype and resistance was evaluated (Kojima *et al.* 2019). In the present study, additional resistance locus was detected on chromosome arm 7AS by GWAS, and many accessions showing stable resistance to pathotypes I and III possessed the resistance haplotype on 7AS (Tables 4, 5). In particular, all 18 accessions that carried the resistance haplotype on 5AL, in addition to those on 7AS, were completely uninfected with pathotype I during the two seasons (Table 6). These 18 accessions were among the 34 resistant to at least pathotype I (Table 2), accounting for approximately half of the resistant varieties (Supplemental Table 8). This result suggests that an interaction between 5AL and 7AS confers robust resistance against pathotype I.

The effects of the three loci, 2DL, 3BS, and 5AL, on infection with pathotype II in JWC also appeared to be consistent with those of a previous report (Supplemental Table 9; Kojima *et al.* 2019). There was also little clear effect of the 7AS locus on the resistance to pathotype II. We then focused on two additional loci, 2AS and 6DS, which were identified in winter wheat varieties bred in the Hokkaido region (Kawaguchi *et al.* 2024, Yamashita *et al.* 2020). The relationship between the 2AS locus and resistance to WYMV pathotypes was difficult to estimate because 86 of 96 accessions possessed the 2AS resistance haplotype. Regarding the 6DS locus, few accessions have the resistance haplotype located on 6DS without also having the haplotypes of 2DL and 3BS, making it difficult to evaluate the effect of the 6DS locus alone on viral infection. However, 13 of 20 accessions carrying both 3BS and 6DS were not infected with pathotype II (Supplemental Table 8). Furthermore, among the 34 accessions resistant to pathotype II, 13 were found to have the resistant haplotypes on both 3BS and 6DS (Supplemental Table 8). These results indicate that the 6DS locus influences the resistance to pathotype II, which appears to be more effective in combination with the 3BS locus.

## Discussion

In this study, we comprehensively investigated the responsiveness of JWC to distinct pathotypes of WYMV. Additionally, we analyzed the distribution of resistance genes and explored the historical acquisition of resistance to WYMV throughout the course of wheat breeding in Japan. Clarifying the categories of incorporated WYMV resistance genes and discerning their effects on individual WYMV pathotypes is anticipated to offer valuable insights for prospective breeding endeavors and the exploration of novel resistance gene candidates.

Through GWAS we detected more than 200 MTAs for WYMV resistance with *p* values ≤0.001 (Fig. 3, Supplemental Tables 6, 7). The most significant MTAs were responsible for pathotype I and were located around the 537–539 Mb region on chromosome arm 5AL, which is presumed to be the same locus as *QYm.njau-5A.1*, which was initially identified as a QTL for resistance to the Chinese WYMV pathotype (Zhu *et al.* 2012). Although an association of this locus on 5AL with resistance to the Japanese WYMV pathotype I has been suggested (Kojima *et al.* 2019), our findings support this proposal with statistical genetics analysis. On the other hand, MTAs for both pathotype I and III resistance were stably identified in the 148–178 Mb region on chromosome arm 7AS, where significant MTAs were included in the 172–178 Mb region. A QTL for WSSMV resistance, *Qssm-mtpsa-7A*, was previously identified in the 7AS of emmer wheat (Holtz *et al.* 2017). The sequences of the flanking markers for *Qssm-mtpsa-7A* were positioned around the 141–170 Mb region on the 7AS of CS reference genome, suggesting a relationship between *Qssm-mtpsa-7A* and MTAs for WYMV resistance. Because WSSMV is closely related to WYMV based on their RNA sequences, it is possible that the same gene is responsible for broad-spectrum resistance. The most noteworthy point regarding the 7AS resistance haplotype is that it confers stable resistance to pathotype I when coexisting with the 5AL resistance haplotype (Table 6). Previously, a model of the effect of resistance genes showed that 5AL was partially effective against pathotype I (Kojima *et al.* 2019). The addition of the 7AS resistance haplotype can strengthen this resistance. Based on these findings, the 7AS locus can be considered a novel WYMV resistance locus that should not be ignored. Although multiple studies, including the present one, have demonstrated that the 2DL resistance locus significantly contributes to resistance against pathotypes I and III, the GWAS conducted in this study failed to identify any stable MTAs. This may be attributed to the limited number of accessions carrying the 2DL resistance haplotype—only five out of 96 in the JWC (Table 5, Fig. 2)—which likely resulted in insufficient statistical power to detect significant MTAs. In contrast, although the resistance haplotypes on chromosome arms 3BS and 6DS were present in 66 and 26 accessions, respectively, indicating relatively high frequencies within the JWC (Table 5, Fig. 2), no MTAs were detected on these loci in the GWAS analysis (Fig. 3, Supplemental Tables 6, 7). The *Ym2* gene, located on chromosome arm 3BS and encoding a CC-NBS-LRR protein, was recently identified as a candidate gene for *Qym2* (Mishina *et al.* 2023). According to this report, nine accessions in the JWC—JWC26, JWC44, JWC51, JWC59, JWC79, JWC86, JWC89, JWC90, and JWC94—carry the resistance-type *Ym2* allele associated with the 3BS resistance haplotype. However, most of these accessions were found to be susceptible to viral infection in the Utsunomiya and Yanagawa experimental fields (Supplemental Table 2). These results suggest that the 3BS resistance locus has only minor effects against pathotypes I and III. Although research on the identification of candidate genes at the 6DS locus remains limited, similar to the 3BS locus, it is likely to exert only a minor effect on resistance to pathotypes I and III.

**Fig. 4.**
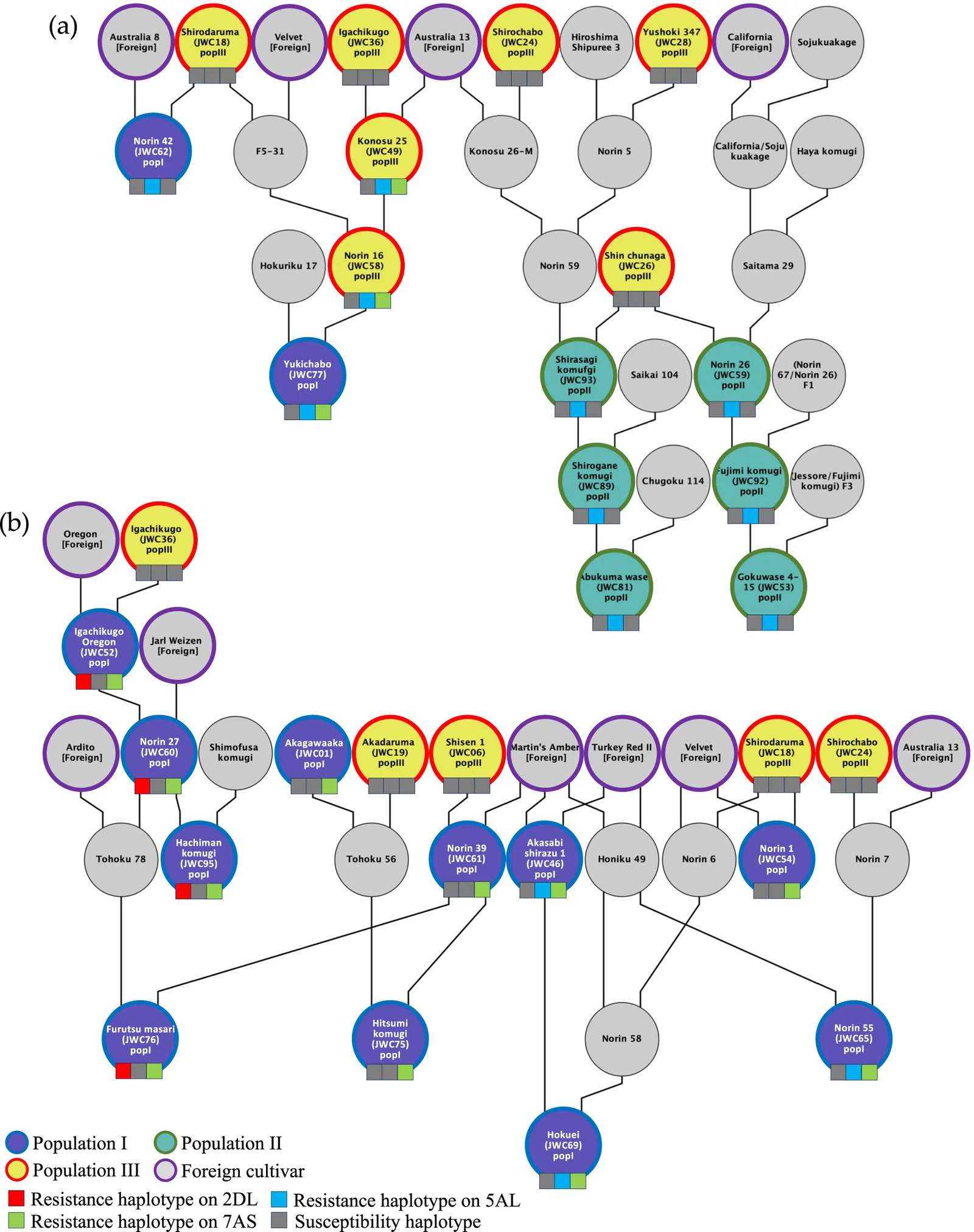
Introduction and propagation of resistance haplotypes in JWC accessions. The pedigree represents the early stages of wheat breeding history in Japan: (a) Central and western Japan and Kyushu region, (b) northern Japan. Pedigree of accessions belonging to population I, II and III and their related accessions is based on Mizuno *et al.* (2024). A detailed pedigree including other JWC accessions is shown in Supplemental Figs. 1 and 2.

Three resistance haplotypes on 2DL, 5AL, and 7AS were mainly distributed in the breeders lines rather than in the landraces and pure selected lines (Fig. 2, Supplemental Table 5), suggesting that these loci were introduced from foreign germplasm. Previous pedigree analyses have estimated that several foreign varieties are part of the origins of WYMV resistance in Japanese wheat varieties (Oda and Kashiwazaki 1989). A recent study showed that JWC accessions can be divided into three groups by genomic structure analysis: populations I, II, and III (Mizuno *et al.* 2024). All accessions in population II comprised modern varieties from the Kanto to Kyushu regions possessed the 5AL resistance haplotype (Fig. 4a, Supplemental Table 2, Supplemental Fig. 1). Mizuno *et al.* (2024) hypothesized that two foreign varieties, ‘California’ and ‘Australia 13’, contributed the formation of genetic characteristics of population II. The allele of *Vrn-A1* conferring a spring growth habit was one of the genetic elements introduced from ‘California’ and ‘Australia 13’ into traditional Japanese varieties (Gotoh 1979). *Vrn-A1* and *wmc415* marker for the 5AL haplotype located in the 589.3 and 535.8 Mb regions on chromosome 5A of CS, respectively (Zhu *et al.* 2021). These findings suggest that the 5AL resistance haplotype may have been introduced simultaneously with *Vrn-A1*. Other accessions carrying the 5AL resistance haplotype in populations I and III also had foreign varieties, such as ‘Champion White’, ‘Turkey Red’, ‘Martin’s Amber’, ‘Australia 8’ and ‘Velvet’ in their pedigree (Fig. 4, Supplemental Figs. 1, 2). Regarding the origin of the resistance haplotype in 2DL, Chen *et al.* (2023) proposed that it originated from a variety in Europe and then entered Japanese varieties. The pedigree of JWC accessions carrying the 2DL resistance haplotype indicates that this haplotype was introduced via USA varieties. ‘Igachikugo Oregon’ (JWC52) is involved in the lineage of these accessions as a common ancestor (Fig. 4b, Supplemental Fig. 2). The USA variety ‘Oregon’ is one of the parents of JWC52 and is presumed to be the source of the 2DL resistance haplotype. Another accession, ‘Dawson 1’ (JWC03), is also a pure selected line of the USA variety ‘Dawson’ (Amano 2000). The distribution of the 7AS resistance haplotype in the JWC pedigree indicated that its origins were not only the foreign varieties mentioned above, but also classical varieties in Hokkaido that had been introduced from the USA (Amano 2000) (Fig. 4b, Supplemental Fig. 2). These foreign germplasms were initially sources of desirable traits, such as adaptability, abiotic stress tolerance, and disease resistance, and eventually contributed to the introduction and accumulation of resistance haplotypes exhibiting WYMV resistance. In northern Japan, foreign varieties have been actively introduced into breeding programs to develop varieties with improved cold tolerance and resistance to snow mold (Fukunaga and Inagaki 1985, Hoshino and Seko 1996). The higher frequencies of resistance haplotypes in 2DL, 5AL, and 7AS in northern Japan reflect this breeding history.

Two resistant haplotypes, 3BS and 6DS, were initially identified as QTLs conferring resistance against pathotype II, *Qym2* and *Qym4*, respectively (Suzuki *et al.* 2015, Yamashita *et al.* 2020). Therefore, the effect of these loci on the resistance to pathotype II was also supported in this study, and their effect seemed to be more pronounced when the two resistance haplotypes accumulated (Supplemental Table 8). These resistant haplotypes were widely distributed among the landraces and pure selected lines (Supplemental Tables 2, 5). Pedigree of ‘OW104’, a source of *Qym4*, contains several traditional varieties of JWC accession carrying the resistance haplotype on 6DS: JWC06, JWC21, JWC24 and JWC36 (Osanai 2010, Osanai *et al.* 2005). Given the sequence diversity of the *Ym2* gene in the 3BS locus (Mishina *et al.* 2023), comprehensive sequence analysis is warranted; nevertheless, the resistance haplotype on 3BS appeared to be retained in the breeders lines. Considering that the resistant haplotypes on 3BS and 6DS originally existed in traditional Japanese wheat, the resistance conferred by 3BS and 6DS may have been effective against some viruses potentially related to WYMV pathotype II that existed in Japan in the past but may have been broken down by emerging viruses such as WYMV pathotype I. This can be inferred from the high frequency of accessions resistant to pathotype II among the traditional varieties from the Kanto to Kyushu regions (Supplemental Table 4). Indeed, among these accessions, some had been identified as resistant to WYM disease in 1933: JWC08, JWC20, JWC21, JWC22, JWC26, and JWC40 (Bokura 1933).

The first case of WYM disease in Japan was reported in Shizuoka, Chubu region, around 1898 (Shizuoka Agricultural Experiment Station 1916). Since the 1910s, this disease has severely damaged wheat production, particularly in the Kanto, Kinki, and Kyushu regions (Bokura 1933). A simultaneous survey by the Ministry of Agriculture and Forestry in 1933 reported that the disease was distributed in 29 of the 47 prefectures from Tohoku to Kyushu, and a subsequent survey in 1938 showed that it had spread to 33 prefectures (Bokura 1933, Ikata and Kawai 1940). It is assumed that the increase in the release of resistant varieties in JWC in the late 1930s was due to this historical background. The distribution of resistance haplotypes on 5AL and 7AS roughly coincides with the release of these resistant varieties (Supplemental Table 2). There were also a few accessions carrying resistance haplotype on 2DL among the JWC accessions (Fig. 2, Supplemental Tables 2, 5). However, 5AL and 7AS were selected for subsequent breeding of resistant varieties. Since the 1980s, the WYM disease has spread again across Japan (Ohto 2005). Although several resistant varieties had been released since the 1950s (Supplemental Table 2), a susceptible variety, ‘Norin 61’ (JWC66, released in 1944), had been the main variety cultivated for over 50 years from the Kanto to Kyushu regions, which is one of the reasons for the broad distribution of pathotype I (Kojima *et al.* 2019, Ohto 2005). As previously reported, pathotype III has emerged in Fukuoka, Kyushu region, as a virulent mutant of pathotype I, which is presumed to have overcome the resistance associated with chromosome 5AL (Kojima *et al.* 2019, Ohto *et al.* 2006). In Iwate, part of the Tohoku region, the resistant varieties with 2DL had been released since the late 1930s, but WYM disease exploded in the late 1980s (Mikoshiba *et al.* 1988). This expansion was driven by the cultivation of ‘Nanbu komugi’ (JWC85), which was a leading variety at that time. A similarly widespread WYMV in the Hokkaido region had also been occurred in the 1990s (Kojima *et al.* 2019, Kusume *et al.* 1997). The leading varieties in northern Japan possessed both resistance haplotypes; i.e., on 5AL and 7AS (Fig. 2, Supplemental Table 2), and this genotype also reflects that infection of the pathotype II had spread. Although several genetics studies have revealed that the 2DL locus has a large effect on the WYMV resistance (Kojima *et al.* 2015, Nishio *et al.* 2010, Suzuki *et al.* 2015), it has only recently been used in breeding program (Kiribuchi-Otobe *et al.* 2019, Kobayashi *et al.* 2020b, Suzuki *et al.* 2022).

Eight JWC accessions exhibited stable resistance, which could not be explained by the five loci 2DL, 3BS, 5AL, 6DS, and 7AS (Supplemental Table 2). ‘Honkei 275’ (JWC47) and ‘Takune komugi’ (JWC72) are resistant to both pathotypes I and III. Their resistance is presumed to be due to the resistance haplotype on 2AS, given that ‘Hokkai 240’ (JWC48), a source of *Q.Ymhk*, is involved in their lineage (Supplemental Fig. 2). Although it cannot be ruled out that the resistance of the other six accessions derives from the 2AS locus, there are other possible causes of resistance. ‘Aso zairai’ (yuubou kappu) (JWC41), ‘Norin 10’ (JWC57) and ‘Norin 16’ (JWC58) showed resistance against both pathotypes I and III and carried multiple resistance haplotypes; JWC41 carries 3BS, 6DS and 7AS, and JWC57 and JWC58 carry 3BS, 5AL and 7AS. This accumulation may contribute to stable resistance. Regarding resistance to WSSMV, two resistance-conferring QTLs, *Qssm-mtpsa-7A* and *Qssm-mtpsa-7B*, were identified in emmer wheat, each of which explained 5–9% of the phenotypic variation, but plants with the two resistance alleles had much stronger resistance with 22–43% of the variance (Holtz *et al.* 2017). The verification of such epistatic interactions among the resistance loci of WYMV is necessary in future studies. As for other varieties—‘Koshigun zairaishu’ (JWC23), ‘Hiroshima Shipree’ (JWC29), and ‘Norin 1’ (JWC54)—only the 3BS or 7AS haplotypes are present, which suggests the presence of unknown resistance genes. Among these accessions, two landraces, JWC23 and JWC41, showed robust resistance in the Utsunomiya, Yanagawa, and Matsumoto fields, although many traditional varieties were susceptible (Supplemental Table 2). The resistance mechanisms in these accessions appear to contribute to durable and broad resistance; therefore, understanding these mechanisms can potentially lead to a breakthrough for conferring robust resistance in breeding programs.

The field experiments in this study showed that the number of infected accessions in both the Utsunomiya and Yanagawa fields differed between the two growing seasons (Table 1). The severity of WYMV infection can vary from year to year owing to environmental factors such as temperature, humidity, and virus density. According to verification of temperature effect on WYMV infection, the daily mean soil temperatures ranged from 8˚C to 15˚C and the optimal soil temperature was from 10˚C to 13˚C. Optimal temperature (air and soil temperature) for the propagation and movement of WYMV in wheat was reported to be 10˚C (Ohto 2005). In Kurume, near Yanagawa, the average temperature during the test period was lower in the second season (2017–2018; Supplemental Fig. 3). The number of infected accessions increased during this season, suggesting that the temperature conditions during 2017–2018 were close to the optimum temperature for WYMV activity. The average temperature of Utsunomiya was also slightly lower during the second season (Supplemental Fig. 3). Although there were infections in the first season but not in the second season, the number of infected accessions slightly increased in the second season (Table 1). Conversely, these findings indicate that a warm winter season, when temperatures do not drop to the optimal level, suppresses the behavior of WYMV and makes it less likely for the disease to occur. In general, the temperature sensitivity of wheat plants may influence the prevalence of WYM disease (Ohki *et al.* 2014). As global warming leads to an increase in warm winters, the risk of misjudging the inherent reactivity of wheat to WYMV may increase, leading to sudden WYMV outbreaks. Conferring robust resistance can avoid this risk; therefore, it is important to introduce effective and durable resistance genes into wheat varieties. Having the resistance haplotype on 2DL appears to be necessary to acquire robust resistance to WYMV (Table 5, Supplemental Tables 2, 8, 9; Kojima *et al.* 2019). One of the resistance accessions with this locus, ‘Igachikugo Oregon’ (JWC52), was developed in 1914 and has long been known as a resistance variety for over 80 years (Bokura 1933). This indicates that the resistance gene on 2DL has superior durability in conferring protection against WYM disease. In support of this, two genes encoding papain-like cysteine protease (TaRD21A) and CC-NBS-LRR (Ym1), which are associated with WYM disease resistance, were identified as candidates for the 2DL QTL (Chen *et al.* 2025, Liu *et al.* 2023). This genomic region may contain multiple resistance genes resulting in broad-spectrum resistance. In recent genomic studies, researchers have inferred that the resistance haplotype on 2DL originated in a wild wheat relative (Chen *et al.* 2023, Kobayashi *et al.* 2020a). This may have introduced a defective trait by linkage drag, which is unfavorable for breeding. Breeding lines that improve defective traits have been developed, making the resistance haplotype on 2DL more accessible (Kobayashi *et al.* 2020b). Additionally, the 2AS resistance haplotype is expected to have a significant effect on broad-spectrum resistance in Japanese WYMV pathotypes (Kawaguchi *et al.* 2024). In this study, we propose a new combination of resistance haplotypes that are effective against WYMV: the loci 5AL and 7AS for stable resistance to pathotype I and partial resistance to pathotype III, and 3BS and 6DS for enhanced resistance to pathotype II. This updated model can contribute to future breeding strategies: the resistance haplotype on 2DL is the most effective in all areas of Japan; the introduction of resistance haplotypes of both the 3BS and 6DS loci in addition to 2DL is effective in northern Japan where pathotype II is prevalent, and the introduction of both 5AL and 7AS haplotypes instead of 2DL is also effective in central and western Japan where pathotype I is distributed; thus, it seems advisable to accumulate 7AS resistance haplotype in addition to 2DL in areas where pathotype III is occurring. Furthermore, the search in traditional varieties for as yet unknown genes that confer resistance and analysis of epistatic interactions among infection-resistant genes are promising research topics that deserve attention in wheat crop improvement programs.

Through research using the JWC variety set, we surveyed the behavior of resistance genes in the history of Japanese wheat breeding and obtained useful information for future breeding of resistant wheat varieties. In an ever-changing agricultural environment, the development of crops resistant to soil-borne viral diseases such as WYM disease is extremely important. Therefore, it is important to study the dynamics of multiple resistance genes.

## Author Contribution Statement

ZN, FK, and MF conceived of the study. SO analyzed the phenotype and genotype data. HK, KK, MF, and HM conducted phenotypic surveys. FK and ZN were responsible for genotyping. FK, SO, and ZN drafted the manuscript.

## Supplementary Material

Supplemental Figures

Supplemental Tables

## Figures and Tables

**Table 1.** Field trials of response of Japanese wheat core collection (JWC) accessions to pathotypes I and III of wheat yellow mosaic virus (WYMV)

Experimental field	Tested season	Number of accessions		
		Resistant	Susceptible	PRV*^a^* (%)
Utsunomiya Pathotype I	2016–2017	40	56	41.7
	2017–2018	37	59	38.5
	Stable resistance	33	–	34.4
Yanagawa Pathotype III	2016–2017	19	77	19.8
	2017–2018	12	84	12.5
	Stable resistance	12	–	12.5

*^a^* Percentage of varieties that are resistant to WYMV.

**Table 2.** Response patterns of JWC accessions to WYMV pathotypes

Pathotype I	Pathotype III	Number of accessions	Non-infection with pathotype II
Resistant	Resistant	12*^a^*	6
Resistant	Susceptible	22	4
Susceptible	Resistant	0	0
Susceptible	Susceptible	62	29
Total		96	39

*^a^* ‘Norin 1’ (JWC54) is included.

**Table 3.** Geographical and historical distribution of JWC accessions, excluding cv. ‘Chinese Spring’, in response to pathotypes I and III

Area	Category	Pathotype I				Pathotype III		
		R*^a^*	S*^a^*	PRV*^b^* (%)		R	S	PRV (%)
Northern Japan (n = 32)	Landraces, pure-selected lines	2	6	25.0		0	8	0.0
	Breeders lines	18	6	75.0		8	16	33.3
	Total	20	12	62.5		8	24	25.0
Central and western Japan (n = 47)	Landraces, pure-selected lines	2	24	7.7		1	25	3.8
	Breeders lines	10	11	47.6		2	19	9.5
	Total	12	35	25.5		3	44	6.4
Kyushu (n = 16)	Landraces, pure-selected lines	1	9	10.0		1	9	10.0
	Breeders lines	1	5	16.7		0	6	0.0
	Total	2	14	12.5		1	15	6.3
Total (n = 95)	Landraces, pure-selected lines	5	39	11.4		2	42	4.5
	Breeders lines	29	22	56.9		10	41	19.6
	Total	34	61	35.8		12	83	12.6

*^a^* ‘R’ and ‘S’ denote ‘Resistant’ and ‘Susceptible’ to each WYMV pathotype, respectively. *^b^* Percentage of varieties that are resistant to WYMV.

**Table 4.** Haplotype analysis of the WYMV resistance marker *G_Chr7A_173288218*

Haplotype	Number of accessions	Pathotype I				Pathotype III		
		R*^a^*	S*^a^*	PRV*^b^* (%)		R	S	PRV (%)
C (ref)	60	8	52	13.3		1	59	1.7
T (alt)	36	26	10	72.2		11	25	30.6

*^a^* ‘R’ and ‘S’ denote ‘Resistant’ and ‘Susceptible’ to each WYMV pathotype, respectively. *^b^* Percentage of varieties that are resistant to WYMV.

**Table 5.** Effect of each virus-resistant haplotype on response to pathotypes I and III

Resistant haplotype	Number of accessions	Pathotype I				Pathotype III		
		R*^a^*	S*^a^*	PRV*^b^* (%)		R	S	PRV (%)
2DL	5	5	0	100		4	1	80.0
3BS	67	19	48	28.4		7	60	10.4
5AL	33	24	9	72.7		4	29	12.1
6DS	26	5	21	19.2		3	23	11.5
7AS	36	26	10	72.2		11	25	30.6

*^a^* ‘R’ and ‘S’ denote ‘Resistant’ and ‘Susceptible’ to each WYMV pathotype, respectively. *^b^* Percentage of varieties that are resistant to WYMV.

**Table 6.** Effect of haplotype combination of resistance-conferring loci 5AL and 7AS on response to pathotypes I and III

Genotype*^a^*		Number of accessions	Pathotype I				Pathotype III		
5AL	7AS		R*^b^*	S*^b^*	PRV*^c^* (%)		R	S	PRV (%)
R	R	18	18	0	100		4	14	22.2
R	S	15	6	9	40.0		0	15	0
S	R	18	8	10	44.4		7	11	38.9
S	S	45	2	43	4.4		1	44	2.2
Total		96	34	62	35.4		12	84	12.5

*^a^* ‘R’ and ‘S’ in genotype column denote ‘Resistant’ and ‘Susceptible’ haplotypes on each locus, respectively. *^b^* ‘R’ and ‘S’ denote ‘Resistant’ and ‘Susceptible’ against each WYMV pathotype, respectively. *^c^* Percentage of varieties resistant to WYMV.
