# Supplementary material for: Dynamics of resistant genes and their relationship to resistance to wheat yellow mosaic disease in Japanese wheat germplasms
Source: Breed Sci. 2026 Apr 21;76(2):165–79. doi: 10.1270/jsbbs.25058 (PMC13494331; doi:10.1270/jsbbs.25058)
Supplement: Supplementary file 1 [file 76_165_s1.pdf]

Kobayashi et al./Breeding Science
doi: 10.1270/jsbbs.25058

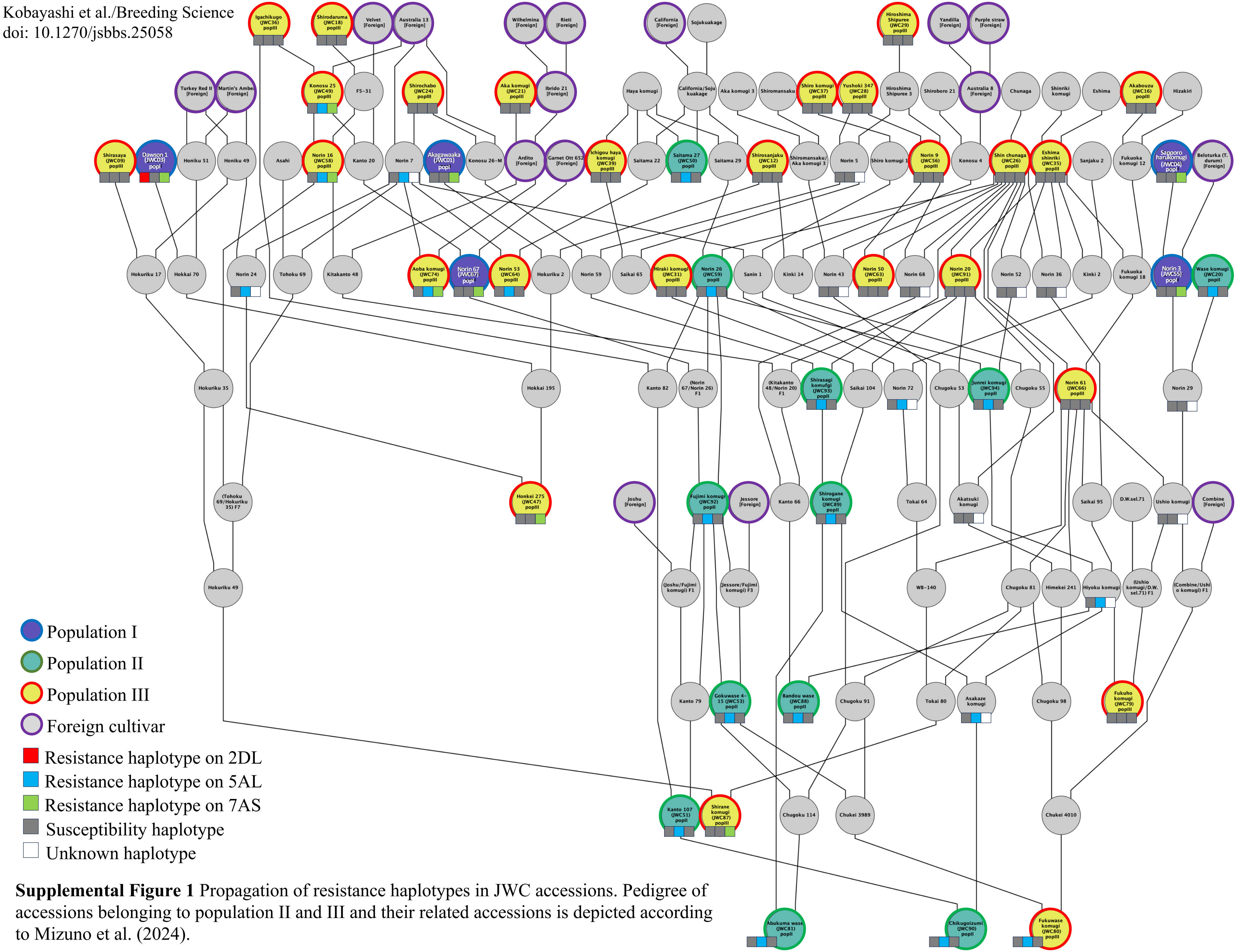

Supplemental Figure 1 Propagation of resistance haplotypes in JWC accessions. Pedigree of accessions belonging to population II and III and their related accessions is depicted according to Mizuno et al. (2024).

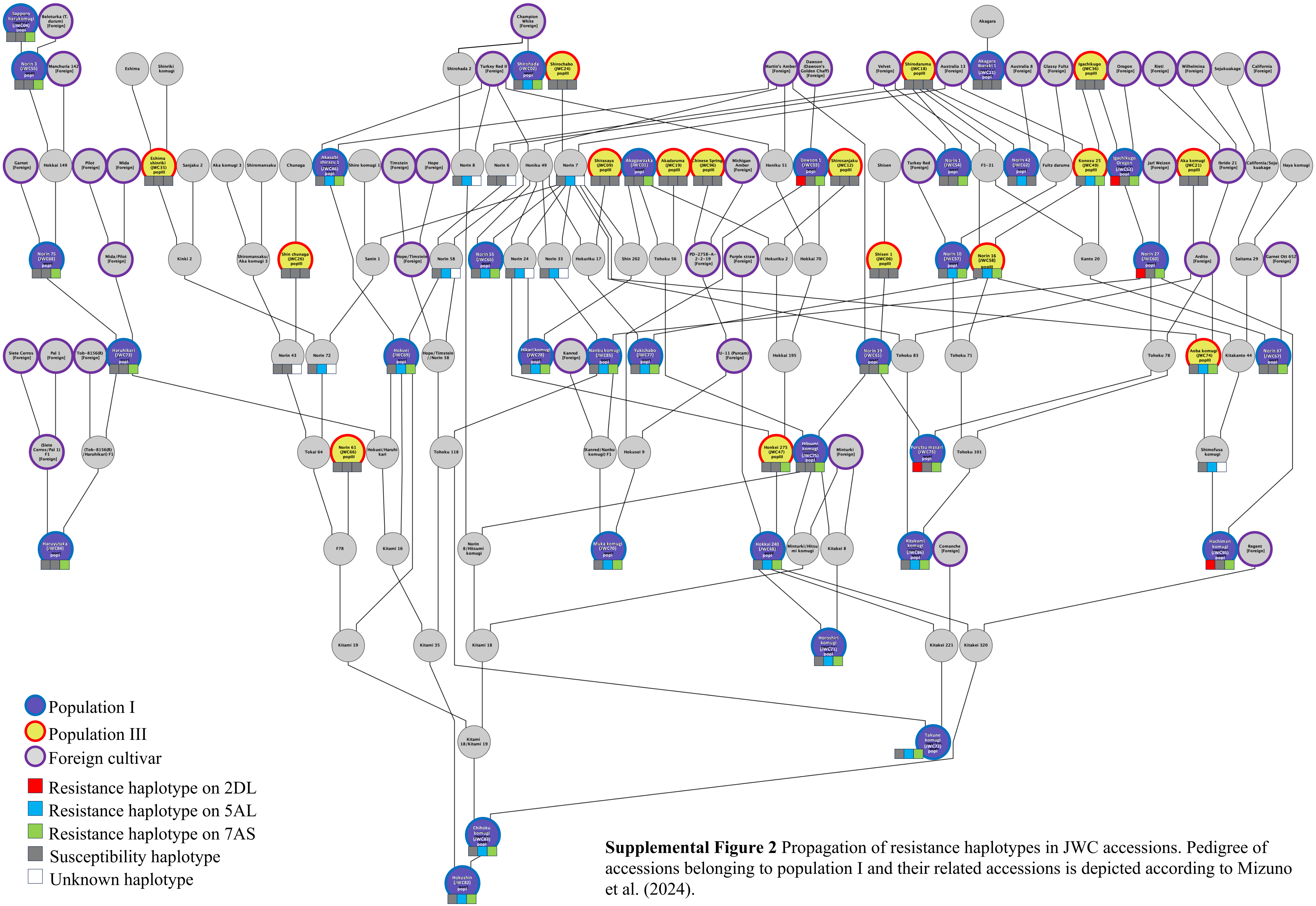

Supplemental Figure 2 Propagation of resistance haplotypes in JWC accessions. Pedigree of accessions belonging to population I and their related accessions is depicted according to Mizuno et al. (2024).

(a)

(b)

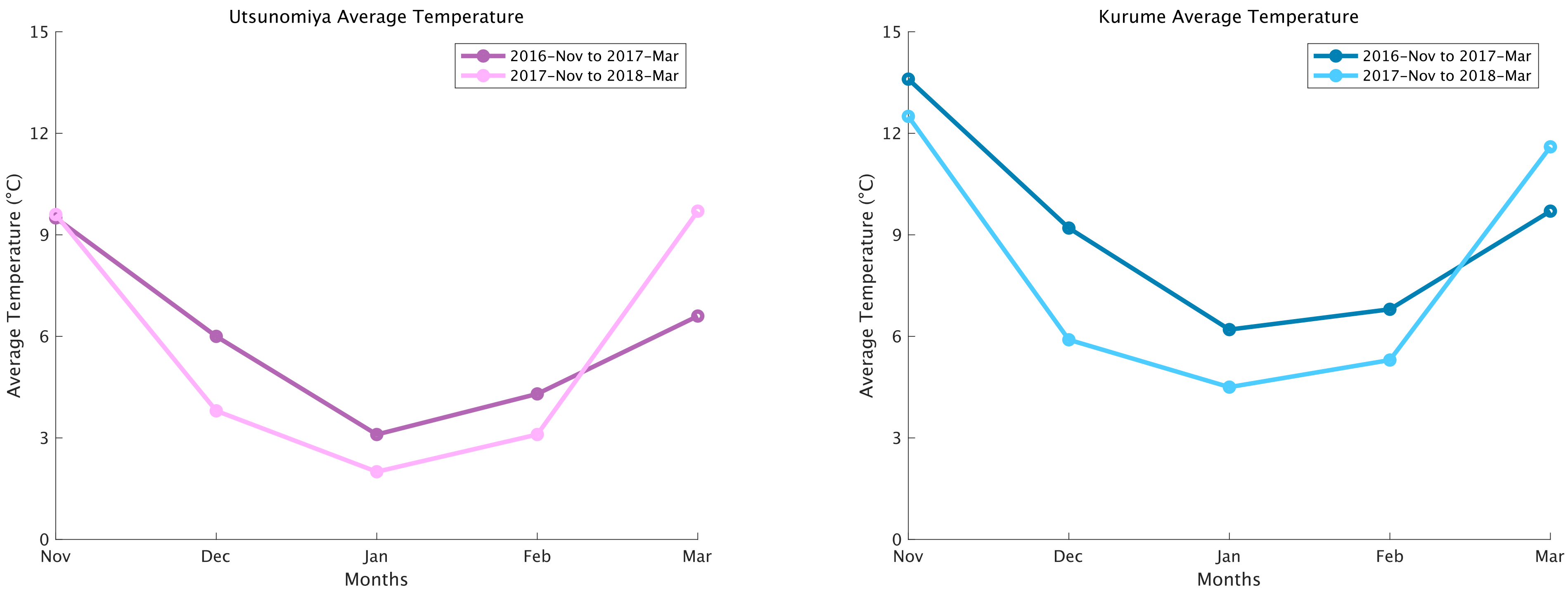

Supplemental Figure 3 Monthly mean temperature between 2016-2017 and 2017-2018 seasons at two locations. Data in Utsunomiya (a) and Kurume (b) near Yanagawa were obtained from the Japan Meteorological Agency.
