# Supplementary material for: Dynamics of resistant genes and their relationship to resistance to wheat yellow mosaic disease in Japanese wheat germplasms
Source: Breed Sci. 2026 Apr 21;76(2):165–79. doi: 10.1270/jsbbs.25058 (PMC13494331; doi:10.1270/jsbbs.25058)
Supplement: Supplementary file 2 [file 76_165_s2.pdf]

Kobayashi et al./Breeding Science
doi: 10.1270/jsbbs.25058

Supplemental Table 1. Primer information used in this study

Chromosome arm	Marker name	Primer	Sequence	Annealing temperature	Reference
2AS	*snp4212*	FAM	5'-gaaggtgaccaagttcatgctATGGTCATCCCAATATGCGC-3'	-	Kawaguchi *et al*. (2024)
		HEX	5'-gaaggtcggagtcaacggattAATGGTCATCCCAATATGCGT-3'		
		COM	5'-TAATGTTTAGCTTTTCTAATACAATGATAG-3'		
2DL	*SPT20446*	Forward	5'-CTTAACACGAGAAGCTTGCA-3'	60 °C	Kobayashi *et al.* (2020)
		Reverse	5'-TTATTCGAGGGAAAACTGGT-3'		
3BS	*wmc754*	Forward	5'-ATCCACATGAACCTCAACTTATGG-3'	55 °C	Suzuki *et al.* (2015)
		Reverse	5'-GGCATTGTTGTTGTACTGCAGTC-3'		
5AL	*wmc415*	Forward	5'-AATTCGATACCTCTCACTCACG-3'	55 °C	Somers *et al*. (2004)
		Reverse	5'-TCAACTGCTACAACCTAGACCC-3'		
6DS	*cfd49*	Forward	5'-TGAGTTCTTCTGGTGAGGCA-3'	55 °C	Yamashita *et al*. (2020)
		Reverse	5'-GAATCGGTTCACAAGGGAAA-3'		

Supplemental Table 2 Information of JWC accessions, their response to the WYMV pathotypes evaluated by ELISA test and their genotypes of WYMV resistance loci

ID	Cultivar name	Category [a]	Cultivated period or established year [b]	Origin			Pathotype I [c] Utsunomiya		Pathotype III [c] Yanagawa		Pathotype II [c] Obihiro	Genotypes of markers associated WYMV resistance QTLs [d]					
				Area	Region	Prefecture	2016-2017	2017-2018	2016-2017	2017-2018	2022-2023	2AS *snp4212*	2DL *SPT20446*	3BS *wmc754*	5AL *wmc415*	6DS *cfd49*	7AS *G_Chr7A_173288218*
JWC 01	AKAGAWA AKA	landraces, pure-selected lines	1906	northern Japan	Hokkaido	Hokkaido	+	+	+	+	+	S	S	S	S	S	R
JWC 02	SHIRO HADA	landraces, pure-selected lines	1924	northern Japan	Hokkaido	Hokkaido	–	–	+	+	+	S	S	R	R	S	R
JWC 03	DAWSON 1	landraces, pure-selected lines	1923	northern Japan	Hokkaido	Hokkaido	–	–	+	+	nc	S	R	S	S	R	R
JWC 04	SAPPORO HARU KOMUGI	landraces, pure-selected lines	1906	northern Japan	Hokkaido	Hokkaido	–	+	+	+	+	R	S	R	S	R	R
JWC 05	SOUSHUU 2	landraces, pure-selected lines	1931	northern Japan	Tohoku	Aomori	+	+	+	+	+	R	S	S	S	R	S
JWC 06	SHISEN 1	landraces, pure-selected lines	1920	northern Japan	Tohoku	Iwate	+	+	+	+	–	R	S	R	S	R	S
JWC 07	ZAIRAI FLUTZ	landraces, pure-selected lines	-	northern Japan	Tohoku	Iwate	+	+	+	+	–	R	S	R	S	U	S
JWC 08	NISHIMURA	landraces, pure-selected lines	1932	northern Japan	Tohoku	Yamagata	+	+	+	+	–	R	S	R	S	U	S
JWC 09	SHIRASAYA	landraces, pure-selected lines	-	central and western Japan	Kanto	Ibaraki	+	+	+	+	nc	R	S	R	S	U	S
JWC 10	FUKOKU	landraces, pure-selected lines	1912	central and western Japan	Kanto	Ibaraki	+	+	+	+	+	R	S	R	S	U	S
JWC 11	AKAGARA IBARAKI 1	landraces, pure-selected lines	1906	central and western Japan	Kanto	Ibaraki	+	+	+	+	+	S	S	R	S	U	S
JWC 12	SHIRO SANJAKU	landraces, pure-selected lines	-	central and western Japan	Kanto	Ibaraki	+	+	+	+	–	R	S	R	S	R	S
JWC 13	AKA BORO 1	landraces, pure-selected lines	1921	central and western Japan	Kanto	Tochigi	+	+	+	+	+	R	S	S	S	U	S
JWC 14	NITTA WASE	landraces, pure-selected lines	-	central and western Japan	Kanto	Gunma	+	+	+	+	–	R	S	R	S	U	S
JWC 15	SUNEKIRI 15	landraces, pure-selected lines	1922	central and western Japan	Kanto	Gunma	+	+	+	+	–	R	S	S	S	R	S
JWC 16	AKA BOUZU	landraces, pure-selected lines	-	central and western Japan	Kanto	Saitama	+	+	+	+	nc	R	S	R	S	U	S
JWC 17	HOSOGARA	landraces, pure-selected lines	1915	central and western Japan	Kanto	Chiba	+	+	+	+	+	S	S	R	S	U	S
JWC 18	SHIRO DARUMA	landraces, pure-selected lines	1932	central and western Japan	Kanto	Kanagawa	+	+	+	+	+	R	S	S	S	U	S
JWC 19	AKA DARUMA	landraces, pure-selected lines	1932	central and western Japan	Kanto	Kanagawa	+	+	+	+	–	R	S	S	S	R	S
JWC 20	WASE KOMUGI	landraces, pure-selected lines	1932	central and western Japan	Kanto	Kanagawa	+	+	+	+	–	R	S	R	R	U	S
JWC 21	AKA KOMUGI	landraces, pure-selected lines	1916	central and western Japan	Chubu	Yamanashi	+	+	+	+	–	R	S	R	S	R	S
JWC 22	SHIBU SHIRAZU	landraces, pure-selected lines	1916	central and western Japan	Chubu	Nagano	+	+	+	+	–	R	S	R	S	R	S
JWC 23	KOSHIGUN ZAIRAISHU	landraces, pure-selected lines	-	central and western Japan	Chubu	Niigata	–	–	–	–	nc	R	S	R	S	U	S
JWC 24	SHIRO CHABO	landraces, pure-selected lines	-	central and western Japan	Chubu	Mie	+	+	+	+	+	R	S	R	S	R	S
JWC 25	SAKOBORE	landraces, pure-selected lines	1932	central and western Japan	Chubu	Shizuoka	+	+	+	+	–	R	S	S	S	U	S
JWC 26	SHIN CHUUNAGA	landraces, pure-selected lines	1925	central and western Japan	Kinki	Hyogo	+	+	+	+	–	R	S	R	S	U	S
JWC 27	HATAKEDA KOMUGI	landraces, pure-selected lines	1919	central and western Japan	Chugoku	Okayama	+	+	+	+	–	R	S	R	S	R	S
JWC 28	YUUSHOUKI 347	landraces, pure-selected lines	-	central and western Japan	Chugoku	Okayama	+	+	+	+	+	R	S	S	S	U	S
JWC 29	HIROSHIMA SHIPUREE	landraces, pure-selected lines	-	central and western Japan	Chugoku	Hiroshima	–	–	–	+	nc	R	S	R	S	U	S
JWC 30	MUBOU CHINKO	landraces, pure-selected lines	1922	central and western Japan	Chugoku	Hiroshima	+	+	+	+	+	R	S	R	S	R	S
JWC 31	HIRAKI KOMUGI	landraces, pure-selected lines	-	central and western Japan	Chugoku	Tottori	+	+	+	+	–	R	S	R	S	R	S
JWC 32	NAKA SOUSHUU	landraces, pure-selected lines	-	central and western Japan	Shikoku	Kagawa	+	+	–	+	nc	R	S	R	S	R	S
JWC 33	HOUMAN	landraces, pure-selected lines	-	central and western Japan	Kanto	Tochigi	+	+	+	+	+	R	S	R	S	U	S
JWC 34	SEKICHIKU 1	landraces, pure-selected lines	1922	central and western Japan	Chubu	Aichi	+	+	+	+	+	R	S	R	S	U	S
JWC 35	ESHIMA SHINRIKI	breeders lines	1913	Kyushu	Kyushu	Fukuoka	+	+	+	+	nc	R	S	R	S	R	S
JWC 36	IGA CHIKUGO	landraces, pure-selected lines	-	Kyushu	Kyushu	Saga	+	–	+	+	–	R	S	R	S	R	S
JWC 37	SHIRO KOMUGI	landraces, pure-selected lines	-	Kyushu	Kyushu	Saga	+	+	+	+	–	R	S	S	S	U	S
JWC 38	CHIKUZEN	landraces, pure-selected lines	-	Kyushu	Kyushu	Saga	+	+	+	+	+	R	S	R	S	U	S
JWC 39	ICHIGOU HAYA KOMUGI	landraces, pure-selected lines	1922	Kyushu	Kyushu	Kumamoto	+	+	+	+	–	R	S	R	S	U	S
JWC 40	SHIRO BUNBU	landraces, pure-selected lines	1916	Kyushu	Kyushu	Kumamoto	+	–	+	+	–	R	S	R	S	U	S
JWC 41	ASO ZAIRAI (YUUBOU KAPPU)	landraces, pure-selected lines	-	Kyushu	Kyushu	Kumamoto	–	–	–	–	–	R	S	R	S	R	R
JWC 42	SOTOME	landraces, pure-selected lines	1923	Kyushu	Kyushu	Nagasaki	+	+	+	+	nc	R	S	R	S	R	S
JWC 43	SADA BOUZU	landraces, pure-selected lines	-	Kyushu	Kyushu	Miyazaki	+	+	+	+	+	R	S	R	S	U	S
JWC 44	NOBEOKABOUZU KOMUGI	landraces, pure-selected lines	-	Kyushu	Kyushu	Miyazaki	+	+	+	+	+	R	S	R	S	U	S
JWC 45	SAKIGAKE 1	landraces, pure-selected lines	1925	Kyushu	Kyushu	Kagoshima	+	+	+	+	–	R	S	R	S	R	S
JWC 46	AKASABI SHIRAZU 1	breeders lines	1917	northern Japan	Hokkaido	Hokkaido	–	–	–	+	+	S	S	S	R	S	R
JWC 47	HONKEI 275	breeders lines	-	northern Japan	Hokkaido	Hokkaido	–	–	–	–	–	R	S	R	S	R	R
JWC 48	HOKKAI 240	breeders lines	1956	northern Japan	Hokkaido	Hokkaido	–	–	–	–	nc	R	S	S	R	S	R
JWC 49	KOUNOSU 25	breeders lines	1937	central and western Japan	Kanto	Saitama	–	–	+	+	nc	R	S	R	R	U	R
JWC 50	SAITAMA 27	breeders lines	1918	central and western Japan	Kanto	Saitama	–	–	+	+	–	R	S	R	R	R	S

JWC 51	KANTOU 107	breeders lines	1984	central and western Japan	Kanto	Ibaraki	–	+	+	+	–	R	S	R	R	U	S
JWC 52	IGA CHIKUGO OREGON	breeders lines	1914	central and western Japan	Chubu	Nagano	–	–	–	–	–	R	R	S	S	R	R
JWC 53	GOKUWASE 4-15	breeders lines	1958	central and western Japan	Chugoku	Hiroshima	–	–	+	+	–	R	S	R	R	U	S
JWC 54	NORIN 1	breeders lines	1929	northern Japan	Tohoku	Iwate	+	–	–	–	+	R	S	S	S	U	R
JWC 55	HARUMAKI KOMUGI NORIN 3	breeders lines	1930	northern Japan	Hokkaido	Hokkaido	+	+	+	+	nc	R	S	S	S	R	R
JWC 56	NORIN 9	breeders lines	1935	central and western Japan	Chubu	Aichi	+	+	+	+	–	R	S	R	S	R	S
JWC 57	NORIN 10	breeders lines	1935	northern Japan	Tohoku	Iwate	–	–	–	–	+	R	S	R	R	S	R
JWC 58	NORIN 16	breeders lines	1936	central and western Japan	Kanto	Gunma	–	–	–	–	+	R	S	R	R	U	R
JWC 59	NORIN 26	breeders lines	1937	central and western Japan	Kinki	Nara	–	–	+	+	–	R	S	R	R	U	S
JWC 60	NORIN 27	breeders lines	1937	northern Japan	Tohoku	Iwate	–	–	–	–	–	S	R	S	S	S	R
JWC 61	NORIN 39	breeders lines	1939	northern Japan	Tohoku	Iwate	+	+	+	+	+	R	S	S	S	U	R
JWC 62	KOUSHITSU KOMUGI NORIN 42	breeders lines	1939	central and western Japan	Kanto	Gunma	–	–	–	+	nc	R	S	S	R	U	S
JWC 63	NORIN 50	breeders lines	1942	central and western Japan	Kanto	Gunma	+	+	+	+	–	R	S	R	S	R	S
JWC 64	NORIN 53	breeders lines	1943	central and western Japan	Chubu	Aichi	–	+	+	+	–	R	S	S	R	U	S
JWC 65	NORIN 55	breeders lines	1943	northern Japan	Tohoku	Iwate	–	–	+	+	+	R	S	S	R	U	R
JWC 66	NORIN 61	breeders lines	1944	Kyushu	Kyushu	Saga	+	+	+	+	nc	R	S	R	S	U	S
JWC 67	NORIN 67	breeders lines	1944	central and western Japan	Kanto	Gunma	+	+	+	+	+	R	S	R	S	U	R
JWC 68	HARUMAKI KOMUGI NORIN 75	breeders lines	1947	northern Japan	Hokkaido	Hokkaido	+	+	+	+	+	R	S	R	S	R	R
JWC 69	HOKUEI	breeders lines	1954	northern Japan	Hokkaido	Hokkaido	–	–	–	+	+	S	S	S	R	S	R
JWC 70	MUKA KOMUGI	breeders lines	1968	northern Japan	Hokkaido	Hokkaido	–	–	+	+	+	S	S	S	R	S	R
JWC 71	HOROSHIRI KOMUGI	breeders lines	1974	northern Japan	Hokkaido	Hokkaido	–	–	+	+	+	R	S	S	R	S	R
JWC 72	TAKUNE KOMUGI	breeders lines	1974	northern Japan	Hokkaido	Hokkaido	–	–	–	–	nc	R	S	S	R	S	R
JWC 73	HARUHIKARI	breeders lines	1965	northern Japan	Hokkaido	Hokkaido	+	+	+	+	nc	R	S	S	S	S	R
JWC 74	AOBA KOMUGI	breeders lines	1951	northern Japan	Tohoku	Iwate	–	–	+	+	+	R	S	R	R	U	R
JWC 75	HITSUMI KOMUGI	breeders lines	1953	northern Japan	Tohoku	Iwate	+	+	+	+	nc	R	S	R	S	S	R
JWC 76	FURUTSU MASARI	breeders lines	1956	northern Japan	Tohoku	Iwate	–	–	–	–	–	R	R	R	S	U	R
JWC 77	YUKICHABO	breeders lines	1952	central and western Japan	Chubu	Niigata	–	–	+	+	nc	R	S	S	R	U	R
JWC 78	HIKARI KOMUGI	breeders lines	1952	central and western Japan	Chubu	Niigata	–	–	+	+	+	R	S	R	R	U	R
JWC 79	FUKUHO KOMUGI	breeders lines	1979	central and western Japan	Kanto	Saitama	+	+	+	+	–	R	S	R	S	U	S
JWC 80	FUKUWASE KOMUGI	breeders lines	1983	central and western Japan	Chugoku	Hiroshima	+	+	+	+	–	R	S	R	R	U	S
JWC 81	ABUKUMA WASE	breeders lines	1992	Kyushu	Kyushu	Fukuoka	–	–	+	+	nc	R	S	R	R	U	S
JWC 82	HOKUSHIN	breeders lines	1995	northern Japan	Hokkaido	Hokkaido	–	–	–	+	+	R	S	S	R	S	R
JWC 83	CHIHOKU KOMUGI	breeders lines	1981	northern Japan	Hokkaido	Hokkaido	–	–	+	+	+	R	S	S	R	S	R
JWC 84	HARUYUTAKA	breeders lines	1985	northern Japan	Hokkaido	Hokkaido	+	+	+	+	+	R	S	S	S	U	R
JWC 85	NANBU KOMUGI	breeders lines	1951	northern Japan	Tohoku	Iwate	–	–	+	+	+	S	S	R	R	U	R
JWC 86	KITAKAMI KOMUGI	breeders lines	1959	northern Japan	Tohoku	Iwate	–	–	–	+	+	R	S	R	R	U	R
JWC 87	SHIRANE KOMUGI	breeders lines	1986	central and western Japan	Chubu	Nagano	+	+	+	+	+	R	S	R	S	U	R
JWC 88	BANDOU WASE	breeders lines	1990	central and western Japan	Kanto	Ibaraki	+	–	+	+	+	R	S	R	R	U	S
JWC 89	SHIROGANE KOMUGI	breeders lines	1974	Kyushu	Kyushu	Fukuoka	–	+	+	+	–	R	S	R	R	U	S
JWC 90	CHIKUGOIZUMI	breeders lines	1993	Kyushu	Kyushu	Fukuoka	–	+	+	+	–	R	S	R	R	U	S
JWC 91	NORIN 20	breeders lines	1935	Kyushu	Kyushu	Saga	+	+	+	+	nc	R	S	R	S	U	S
JWC 92	FUJIMI KOMUGI	breeders lines	1960	central and western Japan	Kanto	Saitama	–	+	+	+	–	R	S	R	R	U	S
JWC 93	SHIRASAGI KOMUGI	breeders lines	1956	central and western Japan	Chugoku	Hiroshima	–	+	+	+	–	R	S	R	R	U	S
JWC 94	JUNREI KOMUGI	breeders lines	1957	central and western Japan	Shikoku	Kagawa	–	–	+	+	–	R	S	R	R	U	S
JWC 95	HACHIMAN KOMUGI	breeders lines	1973	northern Japan	Tohoku	Iwate	–	–	–	–	–	R	R	R	S	U	R
JWC 96	CHINESE SPRING	-	-	-	-	-	+	+	+	+	nc	R	S	R	S	U	S

a Category is based on Kobayashi *et al.* (2016) and Kojima *et al.* (2017).

b 'Cultivated period' for landraces and pure-selected lines and 'established year' for breeders lines

c "+": WYMV detectection, "–": non-detection, "nc": infection not confirmed

d "R": resistance, "S": susceptible, "U": unknown resistant or not

Supplemental Table 3 Reaction of nine accessions to pathotype I in Matsumoto by ELISA test and their genotype of WYMV resistance loci

ID	Cultivar name	Pathotype I [a] Matsumoto				Genotypes of markers associated WYMV resistance QTLs [b]					
						2AS	2DL	3BS	5AL	6DS	7A
		2020-2021	2021-2022	2022-2023	2023-2024	*snp4212*	*SPT20446*	*wmc754*	*wmc415*	*cfd49*	*G_Chr7A_173288218*
JWC 23	KOSHIGUN ZAIRAISHU	–	–	–	–	R	S	R	S	U	S
JWC 41	ASO ZAIRAI (YUUBOU KAPPU)	–	–	–	–	R	S	R	S	R	R
JWC 54	NORIN 1	–	–	–	–	R	S	S	S	U	R
JWC 85	NANBU KOMUGI	–	–	–	–	S	S	R	R	U	R
JWC 79	FUKUHO KOMUGI	–	–	–/+	–/+	R	S	R	S	U	S
JWC 48	HOKKAI 240	–	–	–	–	R	S	S	R	S	R
JWC 66	NORIN 61	N/A	+	+	–	R	S	R	S	U	S
JWC 96	CHINESE SPRING	N/A	N/A	+	+	R	S	R	S	U	S
-	KITAHONAMI	+	+	+	+	N/A	N/A	N/A	N/A	N/A	N/A

a "+": WYMV detection, "–": non-detectection

b "R": resistance, "S": susceptible, "U": unknown resistant or not

JWC85, 79 and 48 were used as standard varieties for WYMV infection (Ohto 2005).

JWC66, JWC96 and 'Kitahonami' were used as controle for susceptible cultivars.

Supplemental Table 4 Geographical and historical distribution of JWC accessions in responsibility to WYMV pathotype II

Area	Category	Resistant	Susceptible	Not determined	PRV [a] (%)
Northern Japan (n=32)	Landraces, pure-selected lines	3	4	1	37.5
	Breeders lines	4	15	5	16.7
	Total	7	19	6	21.9
Central and western Japan (n=47)	Landraces, pure-selected lines	11	10	5	42.3
	Breeders lines	13	5	3	61.9
	Total	24	15	8	51.1
Kyushu (n=16)	Landraces, pure-selected lines	6	3	1	60
	Breeders lines	2	0	4	33.3
	Total	8	3	5	50
Total (n=95)	Landraces, pure-selected lines	20	17	7	45.5
	Breeders lines	19	20	13	37.3
	Total	39	37	19	41.1

a: Percentage of varieties that are resistant to WYMV

Supplemental Table 5 Geographical and historical distribution of resistance and susceptibility haplotypes of WYMV resistance loci in JWC accessions excluding CS

Area	Category	2AS			2DL			3BS			5AL			6DS				7AS		
		R	S	PRH [a] (%)	R	S	PRH (%)	R	S	PRH (%)	R	S	PRH (%)	R	S	U	PRH (%)	R	S	PRH (%)
Northern Japan (n=32)	Landraces, pure-selected lines	5	3	62.5	1	7	12.5	5	3	62.5	1	7	12.5	4	2	2	50	4	4	50
	Breeders lines	19	5	79.2	3	21	12.5	9	15	37.5	13	11	54.2	3	12	9	12.5	24	0	100
	Total	24	8	75	4	28	12.5	14	18	43.8	14	18	43.8	7	14	11	21.9	28	4	87.5
Central and western Japan (n=47)	Landraces, pure-selected lines	24	2	92.3	0	26	0	20	6	76.9	1	25	3.8	10	0	16	38.5	0	26	0
	Breeders lines	21	0	100	1	20	4.8	17	4	81	15	6	71.4	4	0	17	19	7	14	33.3
	Total	45	2	95.7	1	46	2.1	37	10	78.7	16	31	34	14	0	33	29.8	7	40	14.9
Kyushu (n=16)	Landraces, pure-selected lines	10	0	100	0	10	0	9	1	90	0	10	0	4	0	6	40	1	9	10
	Breeders lines	6	0	100	0	6	0	6	0	100	3	3	50	1	0	5	16.7	0	6	0
	Total	16	0	100	0	16	0	15	1	93.8	3	13	18.8	5	0	11	31.3	1	15	6.7
Total (n=95)	Landraces, pure-selected lines	39	5	88.6	1	43	2.3	34	10	77.3	2	42	4.5	18	2	24	40.9	5	39	11.4
	Breeders lines	46	5	90.2	4	47	7.8	32	19	62.7	31	20	60.8	8	12	31	15.7	31	20	60.8
	Total	85	10	89.5	5	90	5.3	66	29	69.5	33	62	34.7	26	14	55	27.3	36	59	37.9

a: Percentage of distribution of haplotype resistant to WYMV

Supplemental Table 6 List of detected MTAs for pathotype I resistance

Chrom	Pos (bp)	Marker	-log10(P)	Year
2B	800,506,284	*R_Chr2B_800506284*	3.188	2016-2017
2B	800,506,491	*R_Chr2B_800506491*	3.188	2016-2017
2B	800,506,538	*R_Chr2B_800506538*	3.188	2016-2017
5A	473,509,503	*G_Chr5A_473509503*	3.332	2017-2018
5A	499,331,202	*R_Chr5A_499331202*	3.053	2017-2018
5A	500,817,479	*G_Chr5A_500817479*	3.053	2017-2018
5A	500,817,608	*G_Chr5A_500817608*	3.053	2017-2018
5A	501,099,415	*G_Chr5A_501099415*	3.053	2017-2018
5A	505,492,429	*G_Chr5A_505492429*	3.485	2017-2018
5A	510,060,804	*G_Chr5A_510060804*	3.485	2017-2018
5A	510,060,815	*G_Chr5A_510060815*	3.485	2017-2018
5A	511,480,533	*tarc1844*	4.114	2017-2018
5A	513,791,404	*R_Chr5A_513791404*	3.363	2017-2018
5A	513,813,820	*G_Chr5A_513813820*	3.363	2017-2018
5A	513,813,835	*G_Chr5A_513813835*	3.363	2017-2018
5A	513,927,879	*G_Chr5A_513927879*	3.363	2017-2018
5A	521,270,651	*G_Chr5A_521270651*	4.086	2016-2017
			3.239	2017-2018
5A	521,397,743	*G_Chr5A_521397743*	4.086	2016-2017
			3.239	2017-2018
5A	529,713,514	*G_Chr5A_529713514*	4.106	2016-2017
			3.840	2017-2018
5A	535,235,757	*G_Chr5A_535235757*	4.106	2016-2017
			3.840	2017-2018
5A	535,235,780	*G_Chr5A_535235780*	4.106	2016-2017
			3.840	2017-2018
5A	535,494,388	*R_Chr5A_535494388*	4.106	2016-2017
			3.840	2017-2018
5A	535,494,597	*R_Chr5A_535494597*	4.106	2016-2017
			3.840	2017-2018
5A	535,691,704	*G_Chr5A_535691704*	4.106	2016-2017
			3.840	2017-2018
5A	535,691,730	*G_Chr5A_535691730*	4.106	2016-2017
			3.840	2017-2018
5A	535,933,636	*G_Chr5A_535933636*	4.106	2016-2017
			3.840	2017-2018
5A	535,933,651	*G_Chr5A_535933651*	4.106	2016-2017
			3.840	2017-2018
5A	535,933,683	*G_Chr5A_535933683*	4.106	2016-2017
			3.840	2017-2018

5A	536,186,369	*G_Chr5A_536186369*	3.650	2016-2017
			4.243	2017-2018
5A	536,189,113	*G_Chr5A_536189113*	4.106	2016-2017
			3.840	2017-2018
5A	536,235,909	*tarc1848_1*	4.106	2016-2017
			3.840	2017-2018
5A	536,235,939	*tarc1848_2*	4.106	2016-2017
			3.840	2017-2018
5A	536,288,733	*G_Chr5A_536288733*	4.106	2016-2017
			3.840	2017-2018
5A	536,288,734	*G_Chr5A_536288734*	4.293	2016-2017
			5.519	2017-2018
5A	536,288,743	*G_Chr5A_536288743*	4.293	2016-2017
			5.519	2017-2018
5A	537,324,773	*tarc0651_1*	5.396	2016-2017
			4.329	2017-2018
5A	537,324,788	*tarc0651_2*	5.396	2016-2017
			4.329	2017-2018
5A	537,415,315	*G_Chr5A_537415315*	5.396	2016-2017
			4.329	2017-2018
5A	537,415,352	*G_Chr5A_537415352*	6.227	2016-2017
			5.947	2017-2018
5A	537,415,381	*G_Chr5A_537415381*	5.396	2016-2017
			4.329	2017-2018
5A	537,615,348	*G_Chr5A_537615348*	6.131	2016-2017
			3.488	2017-2018
5A	537,662,914	*G_Chr5A_537662914*	5.396	2016-2017
			4.329	2017-2018
5A	538,297,228	*G_Chr5A_538297228*	6.131	2016-2017
			3.488	2017-2018
5A	538,297,248	*G_Chr5A_538297248*	6.131	2016-2017
			3.488	2017-2018
5A	538,297,271	*G_Chr5A_538297271*	6.131	2016-2017
			3.488	2017-2018
5A	538,297,278	*G_Chr5A_538297278*	6.131	2016-2017
			3.488	2017-2018
5A	539,222,788	*G_Chr5A_539222788*	6.131	2016-2017
			3.488	2017-2018
5A	539,398,543	*G_Chr5A_539398543*	6.131	2016-2017
			3.488	2017-2018

5A	539,617,632	*G_Chr5A_539617632*	6.131	2016-2017
			3.488	2017-2018
5A	539,617,641	*G_Chr5A_539617641*	6.131	2016-2017
			3.488	2017-2018
5A	539,737,711	*G_Chr5A_539737711*	6.131	2016-2017
			3.488	2017-2018
5A	539,737,733	*G_Chr5A_539737733*	6.131	2016-2017
			3.488	2017-2018
6A	110,111,910	*G_Chr6A_110111910*	3.275	2016-2017
6A	111,076,637	*G_Chr6A_111076637*	3.275	2016-2017
6A	111,076,681	*G_Chr6A_111076681*	3.275	2016-2017
6A	111,092,451	*R_Chr6A_111092451*	3.275	2016-2017
6A	111,129,689	*R_Chr6A_111129689*	3.275	2016-2017
6A	112,176,367	*R_Chr6A_112176367*	3.275	2016-2017
6A	112,176,406	*R_Chr6A_112176406*	3.275	2016-2017
6A	112,319,395	*R_Chr6A_112319395*	3.275	2016-2017
6A	112,319,626	*R_Chr6A_112319626*	3.275	2016-2017
6A	112,326,351	*R_Chr6A_112326351*	3.275	2016-2017
6A	112,749,438	*R_Chr6A_112749438*	3.275	2016-2017
6A	112,749,477	*R_Chr6A_112749477*	3.275	2016-2017
6A	115,223,954	*R_Chr6A_115223954*	3.275	2016-2017
6A	115,285,980	*R_Chr6A_115285980*	3.275	2016-2017
6A	115,660,148	*G_Chr6A_115660148*	3.275	2016-2017
6A	115,751,904	*G_Chr6A_115751904*	3.275	2016-2017
6A	116,925,845	*R_Chr6A_116925845*	3.275	2016-2017
6A	116,926,005	*R_Chr6A_116926005*	3.275	2016-2017
6A	117,254,161	*G_Chr6A_117254161*	3.275	2016-2017
6A	117,254,224	*G_Chr6A_117254224*	3.275	2016-2017
6A	117,254,265	*G_Chr6A_117254265*	3.275	2016-2017
6A	117,254,274	*G_Chr6A_117254274*	3.275	2016-2017
6A	117,285,778	*G_Chr6A_117285778*	3.275	2016-2017
6A	117,757,399	*R_Chr6A_117757399*	3.275	2016-2017
6A	117,757,450	*R_Chr6A_117757450*	3.275	2016-2017
6A	117,757,646	*R_Chr6A_117757646*	3.275	2016-2017
6A	117,757,655	*R_Chr6A_117757655*	3.275	2016-2017
6A	118,173,783	*G_Chr6A_118173783*	3.275	2016-2017
6A	595,414,647	*G_Chr6A_595414647*	3.081	2017-2018
6A	599,007,179	*G_Chr6A_599007179*	4.181	2016-2017
6A	599,008,834	*G_Chr6A_599008834*	4.181	2016-2017
6A	599,172,762	*G_Chr6A_599172762*	4.181	2016-2017

6B	38,181,640	*G_Chr6B_38181640*	3.193	2016-2017
6B	38,183,731	*G_Chr6B_38183731*	3.193	2016-2017
6B	38,183,783	*G_Chr6B_38183783*	3.193	2016-2017
6B	38,205,333	*G_Chr6B_38205333*	3.193	2016-2017
6B	38,796,619	*G_Chr6B_38796619*	3.193	2016-2017
6B	38,796,652	*G_Chr6B_38796652*	3.193	2016-2017
6B	38,796,692	*G_Chr6B_38796692*	3.193	2016-2017
6B	39,755,945	*G_Chr6B_39755945*	3.193	2016-2017
6B	41,388,997	*G_Chr6B_41388997*	3.193	2016-2017
6B	41,389,004	*G_Chr6B_41389004*	3.193	2016-2017
6B	41,389,015	*G_Chr6B_41389015*	3.193	2016-2017
6B	41,389,082	*G_Chr6B_41389082*	3.193	2016-2017
7A	8,073,436	*G_Chr7A_8073436*	4.492	2017-2018
7A	8,806,017	*G_Chr7A_8806017*	5.529	2017-2018
7A	8,887,215	*tarc1947*	3.194	2017-2018
7A	9,038,937	*G_Chr7A_9038937*	3.194	2017-2018
7A	142,267,834	*G_Chr7A_142267834*	3.072	2016-2017
7A	142,895,873	*G_Chr7A_142895873*	3.072	2016-2017
7A	148,204,234	*R_Chr7A_148204234*	3.395	2016-2017
7A	148,204,295	*R_Chr7A_148204295*	3.395	2016-2017
7A	148,204,335	*R_Chr7A_148204335*	3.395	2016-2017
7A	153,332,492	*G_Chr7A_153332492*	3.072	2016-2017
7A	153,332,514	*G_Chr7A_153332514*	3.395	2016-2017
7A	153,638,912	*G_Chr7A_153638912*	3.395	2016-2017
7A	155,706,725	*R_Chr7A_155706725*	3.072	2016-2017
7A	159,877,228	*G_Chr7A_159877228*	3.072	2016-2017
7A	160,022,241	*G_Chr7A_160022241*	3.072	2016-2017
7A	169,273,410	*G_Chr7A_169273410*	3.072	2016-2017
7A	169,273,426	*G_Chr7A_169273426*	3.072	2016-2017
7A	173,212,909	*G_Chr7A_173212909*	3.395	2016-2017
7A	174,421,918	*G_Chr7A_174421918*	3.094	2017-2018
7A	174,565,563	*R_Chr7A_174565563*	3.395	2016-2017
7A	174,565,611	*R_Chr7A_174565611*	3.395	2016-2017
7A	174,591,482	*snp305_1*	3.094	2017-2018
7A	174,591,516	*snp305_2*	3.395	2016-2017
7A	174,951,999	*R_Chr7A_174951999*	3.094	2017-2018
7A	175,597,355	*R_Chr7A_175597355*	3.395	2016-2017
7A	175,599,804	*R_Chr7A_175599804*	3.094	2017-2018
7A	175,966,952	*G_Chr7A_175966952*	3.395	2016-2017
7A	176,716,132	*G_Chr7A_176716132*	3.094	2017-2018
7A	177,991,292	*G_Chr7A_177991292*	3.094	2017-2018

7B	598,623,828	*R_Chr7B_598623828*	3.037	2017-2018
7B	598,623,988	*R_Chr7B_598623988*	3.037	2017-2018
7B	598,624,055	*R_Chr7B_598624055*	3.037	2017-2018
7B	598,795,581	*G_Chr7B_598795581*	3.094	2017-2018
7B	598,795,586	*G_Chr7B_598795586*	3.094	2017-2018
7B	598,801,910	*G_Chr7B_598801910*	3.094	2017-2018
7B	598,864,114	*G_Chr7B_598864114*	3.094	2017-2018
7B	643,054,898	*R_Chr7B_643054898*	3.157	2017-2018
7B	643,054,915	*R_Chr7B_643054915*	3.157	2017-2018
7B	643,054,984	*R_Chr7B_643054984*	3.157	2017-2018
7B	643,055,090	*R_Chr7B_643055090*	3.157	2017-2018
7B	654,725,933	*G_Chr7B_654725933*	5.018	2017-2018
7B	654,860,913	*G_Chr7B_654860913*	3.863	2017-2018
7B	654,991,941	*G_Chr7B_654991941*	3.863	2017-2018
7B	657,597,409	*G_Chr7B_657597409*	3.062	2017-2018

Supplemental Table 7 List of detected MTAs for pathotype III resistance

Chrom	Pos (bp)	Marker	-log10(P)	Year
1A	504,851,856	*R_Chr1A_504851856*	4.114	2017-2018
1B	678,435,798	*G_Chr1B_678435798*	3.022	2016-2017
1B	678,435,798	*G_Chr1B_678435798*	3.605	2017-2018
1D	438,669,644	*snp3547*	3.047	2017-2018
2A	36,522,921	*G_Chr2A_36522921*	3.176	2016-2017
2A	36,522,929	*G_Chr2A_36522929*	3.176	2016-2017
2A	36,522,947	*G_Chr2A_36522947*	3.176	2016-2017
2A	36,522,952	*G_Chr2A_36522952*	3.176	2016-2017
2A	36,523,001	*G_Chr2A_36523001*	3.176	2016-2017
2A	45,343,019	*G_Chr2A_45343019*	4.783	2017-2018
2A	45,518,231	*G_Chr2A_45518231*	3.776	2017-2018
2B	800,506,284	*R_Chr2B_800506284*	3.946	2016-2017
			3.698	2017-2018
2B	800,506,491	*R_Chr2B_800506491*	3.946	2016-2017
			3.698	2017-2018
2B	800,506,538	*R_Chr2B_800506538*	3.946	2016-2017
			3.698	2017-2018
2D	183,852,751	*G_Chr2D_183852751*	3.555	2016-2017
2D	582,768,668	*G_Chr2D_582768668*	3.689	2017-2018
2D	583,125,886	*G_Chr2D_583125886*	3.689	2017-2018
2D	583,125,933	*G_Chr2D_583125933*	3.689	2017-2018
2D	583,125,963	*G_Chr2D_583125963*	3.689	2017-2018
2D	583,977,922	*G_Chr2D_583977922*	3.689	2017-2018
2D	583,977,928	*G_Chr2D_583977928*	3.689	2017-2018
2D	583,977,940	*G_Chr2D_583977940*	3.689	2017-2018
2D	588,375,222	*G_Chr2D_588375222*	3.689	2017-2018
2D	588,382,905	*G_Chr2D_588382905*	3.689	2017-2018
2D	588,382,924	*G_Chr2D_588382924*	3.689	2017-2018
2D	588,382,937	*G_Chr2D_588382937*	3.689	2017-2018
2D	588,432,279	*G_Chr2D_588432279*	3.689	2017-2018
2D	588,432,319	*G_Chr2D_588432319*	3.689	2017-2018
2D	588,432,332	*G_Chr2D_588432332*	3.689	2017-2018
2D	588,432,338	*G_Chr2D_588432338*	3.689	2017-2018
2D	588,432,350	*G_Chr2D_588432350*	3.689	2017-2018
2D	588,505,579	*G_Chr2D_588505579*	3.689	2017-2018
2D	588,505,649	*G_Chr2D_588505649*	3.689	2017-2018
2D	589,150,898	*G_Chr2D_589150898*	3.689	2017-2018
2D	589,150,917	*G_Chr2D_589150917*	3.689	2017-2018

2D	589,591,657	*G_Chr2D_589591657*	3.689	2017-2018
2D	589,591,687	*G_Chr2D_589591687*	3.689	2017-2018
2D	589,946,870	*G_Chr2D_589946870*	3.689	2017-2018
2D	589,946,885	*G_Chr2D_589946885*	3.689	2017-2018
2D	589,946,894	*G_Chr2D_589946894*	3.689	2017-2018
2D	589,946,907	*G_Chr2D_589946907*	3.689	2017-2018
2D	589,946,922	*G_Chr2D_589946922*	3.689	2017-2018
2D	590,669,700	*G_Chr2D_590669700*	3.689	2017-2018
2D	590,669,782	*G_Chr2D_590669782*	3.689	2017-2018
2D	590,680,620	*G_Chr2D_590680620*	3.689	2017-2018
2D	590,680,653	*G_Chr2D_590680653*	3.689	2017-2018
2D	591,515,392	*G_Chr2D_591515392*	3.689	2017-2018
2D	591,515,441	*G_Chr2D_591515441*	3.689	2017-2018
2D	591,515,472	*G_Chr2D_591515472*	3.689	2017-2018
2D	593,129,306	*G_Chr2D_593129306*	3.689	2017-2018
2D	593,129,312	*G_Chr2D_593129312*	3.689	2017-2018
2D	593,129,489	*G_Chr2D_593129489*	3.689	2017-2018
2D	593,201,697	*G_Chr2D_593201697*	3.689	2017-2018
2D	594,080,627	*G_Chr2D_594080627*	3.689	2017-2018
2D	594,080,707	*G_Chr2D_594080707*	3.689	2017-2018
2D	594,080,717	*G_Chr2D_594080717*	3.689	2017-2018
2D	594,136,489	*G_Chr2D_594136489*	3.689	2017-2018
2D	594,136,497	*G_Chr2D_594136497*	3.689	2017-2018
2D	594,952,433	*G_Chr2D_594952433*	3.689	2017-2018
2D	594,952,457	*G_Chr2D_594952457*	3.689	2017-2018
2D	594,952,463	*G_Chr2D_594952463*	3.689	2017-2018
2D	594,984,077	*G_Chr2D_594984077*	3.689	2017-2018
2D	596,096,507	*G_Chr2D_596096507*	3.689	2017-2018
2D	596,096,516	*G_Chr2D_596096516*	3.689	2017-2018
2D	596,096,557	*G_Chr2D_596096557*	3.689	2017-2018
2D	596,096,584	*G_Chr2D_596096584*	3.689	2017-2018
2D	596,457,653	*G_Chr2D_596457653*	3.689	2017-2018
2D	596,739,643	*G_Chr2D_596739643*	3.689	2017-2018
2D	599,619,749	*G_Chr2D_599619749*	3.689	2017-2018
2D	599,866,898	*G_Chr2D_599866898*	3.689	2017-2018
2D	601,754,513	*G_Chr2D_601754513*	3.689	2017-2018
2D	602,834,614	*G_Chr2D_602834614*	3.689	2017-2018
2D	602,834,651	*G_Chr2D_602834651*	3.689	2017-2018
2D	602,857,724	*G_Chr2D_602857724*	3.689	2017-2018
2D	602,857,743	*G_Chr2D_602857743*	3.689	2017-2018
2D	602,857,767	*G_Chr2D_602857767*	3.689	2017-2018

2D	603,257,737	*G_Chr2D_603257737*	3.689	2017-2018
2D	603,925,489	*G_Chr2D_603925489*	3.689	2017-2018
2D	603,925,504	*G_Chr2D_603925504*	3.689	2017-2018
2D	603,925,568	*G_Chr2D_603925568*	3.689	2017-2018
2D	604,149,476	*tarc2370_1*	3.689	2017-2018
2D	604,905,532	*G_Chr2D_604905532*	3.689	2017-2018
2D	604,905,543	*G_Chr2D_604905543*	3.689	2017-2018
2D	604,910,610	*G_Chr2D_604910610*	3.689	2017-2018
2D	604,910,638	*G_Chr2D_604910638*	3.689	2017-2018
2D	609,566,862	*G_Chr2D_609566862*	3.689	2017-2018
2D	609,566,880	*G_Chr2D_609566880*	3.689	2017-2018
2D	609,685,666	*G_Chr2D_609685666*	3.689	2017-2018
2D	609,685,684	*G_Chr2D_609685684*	3.689	2017-2018
2D	609,878,347	*G_Chr2D_609878347*	3.689	2017-2018
2D	614,159,809	*G_Chr2D_614159809*	3.689	2017-2018
2D	614,159,834	*G_Chr2D_614159834*	3.689	2017-2018
2D	614,159,842	*G_Chr2D_614159842*	3.689	2017-2018
2D	615,218,296	*G_Chr2D_615218296*	3.689	2017-2018
2D	615,218,333	*G_Chr2D_615218333*	3.689	2017-2018
2D	615,218,395	*G_Chr2D_615218395*	3.689	2017-2018
2D	616,047,279	*G_Chr2D_616047279*	3.689	2017-2018
2D	616,047,318	*G_Chr2D_616047318*	3.689	2017-2018
2D	616,245,648	*G_Chr2D_616245648*	3.689	2017-2018
2D	616,247,383	*G_Chr2D_616247383*	3.689	2017-2018
2D	618,641,114	*G_Chr2D_618641114*	3.689	2017-2018
2D	618,867,155	*G_Chr2D_618867155*	3.689	2017-2018
2D	618,867,192	*G_Chr2D_618867192*	3.689	2017-2018
2D	618,996,761	*G_Chr2D_618996761*	3.689	2017-2018
2D	618,996,790	*G_Chr2D_618996790*	3.689	2017-2018
2D	619,045,370	*G_Chr2D_619045370*	3.689	2017-2018
2D	619,045,418	*G_Chr2D_619045418*	3.689	2017-2018
2D	619,045,432	*G_Chr2D_619045432*	3.689	2017-2018
2D	619,045,478	*G_Chr2D_619045478*	3.689	2017-2018
2D	619,068,468	*G_Chr2D_619068468*	3.689	2017-2018
2D	619,068,482	*G_Chr2D_619068482*	3.689	2017-2018
2D	619,068,547	*G_Chr2D_619068547*	3.689	2017-2018
2D	619,463,991	*G_Chr2D_619463991*	3.689	2017-2018
2D	619,464,001	*G_Chr2D_619464001*	3.689	2017-2018
2D	619,877,171	*G_Chr2D_619877171*	3.689	2017-2018
2D	619,877,178	*G_Chr2D_619877178*	3.689	2017-2018
2D	619,877,188	*G_Chr2D_619877188*	3.689	2017-2018

2D	620,032,928	*G_Chr2D_620032928*	3.689	2017-2018
2D	620,450,944	*G_Chr2D_620450944*	3.689	2017-2018
2D	620,450,952	*G_Chr2D_620450952*	3.689	2017-2018
2D	621,066,041	*G_Chr2D_621066041*	3.689	2017-2018
2D	621,066,090	*G_Chr2D_621066090*	3.689	2017-2018
2D	621,120,922	*G_Chr2D_621120922*	3.689	2017-2018
3A	12,668,444	*G_Chr3A_12668444*	4.629	2016-2017
			3.572	2017-2018
3A	627,556,886	*G_Chr3A_627556886*	3.733	2017-2018
3A	715,117,180	*G_Chr3A_715117180*	4.082	2016-2017
3A	715,225,683	*G_Chr3A_715225683*	4.082	2016-2017
3A	715,289,254	*G_Chr3A_715289254*	4.082	2016-2017
3A	728,236,439	*G_Chr3A_728236439*	3.725	2017-2018
3A	728,304,454	*G_Chr3A_728304454*	3.014	2017-2018
3A	750,047,441	*G_Chr3A_750047441*	3.373	2017-2018
3A	750,053,595	*G_Chr3A_750053595*	3.373	2017-2018
3A	750,918,342	*G_Chr3A_750918342*	3.373	2017-2018
3B	1,701,801	*G_Chr3B_1701801*	3.425	2017-2018
3B	1,703,225	*G_Chr3B_1703225*	3.425	2017-2018
3B	16,773,817	*G_Chr3B_16773817*	3.083	2017-2018
3B	62,363,115	*G_Chr3B_62363115*	3.204	2017-2018
3B	464,098,831	*G_Chr3B_464098831*	3.359	2017-2018
3B	586,900,320	*G_Chr3B_586900320*	3.689	2017-2018
3B	588,412,888	*G_Chr3B_588412888*	5.492	2016-2017
			4.903	2017-2018
3B	588,562,920	*G_Chr3B_588562920*	5.004	2016-2017
			4.825	2017-2018
3B	588,762,014	*G_Chr3B_588762014*	5.004	2016-2017
			4.825	2017-2018
3B	589,538,390	*G_Chr3B_589538390*	3.689	2017-2018
3B	589,837,647	*G_Chr3B_589837647*	3.229	2017-2018
3B	617,727,740	*G_Chr3B_617727740*	3.198	2017-2018
3B	751,817,864	*G_Chr3B_751817864*	4.221	2017-2018
3B	752,141,685	*G_Chr3B_752141685*	4.221	2017-2018
3B	752,141,713	*G_Chr3B_752141713*	4.221	2017-2018
3B	752,148,429	*G_Chr3B_752148429*	4.221	2017-2018
3B	753,924,084	*snp5014_2*	3.391	2017-2018
3B	753,924,260	*snp5014_3*	3.391	2017-2018
3B	753,924,284	*snp5014_4*	3.391	2017-2018
3B	753,934,680	*G_Chr3B_753934680*	3.391	2017-2018
3B	753,965,889	*G_Chr3B_753965889*	5.290	2017-2018

3B	777,156,429	*G_Chr3B_777156429*	4.015	2016-2017
3B	777,741,453	*G_Chr3B_777741453*	4.015	2016-2017
3B	779,093,591	*G_Chr3B_779093591*	3.045	2016-2017
3B	779,406,239	*G_Chr3B_779406239*	3.037	2016-2017
3B	779,406,269	*G_Chr3B_779406269*	3.037	2016-2017
3D	115,236,275	*G_Chr3D_115236275*	3.429	2017-2018
3D	115,669,222	*G_Chr3D_115669222*	3.429	2017-2018
4A	661,379,396	*G_Chr4A_661379396*	3.193	2017-2018
4A	725,648,292	*G_Chr4A_725648292*	3.041	2017-2018
4A	725,648,315	*G_Chr4A_725648315*	3.041	2017-2018
4A	725,648,323	*G_Chr4A_725648323*	3.041	2017-2018
4A	725,648,336	*G_Chr4A_725648336*	3.041	2017-2018
4A	725,648,362	*G_Chr4A_725648362*	3.041	2017-2018
4A	725,664,963	*G_Chr4A_725664963*	3.041	2017-2018
4A	725,664,988	*G_Chr4A_725664988*	3.041	2017-2018
4A	725,665,016	*G_Chr4A_725665016*	3.041	2017-2018
4A	725,665,070	*G_Chr4A_725665070*	3.041	2017-2018
4A	725,665,077	*G_Chr4A_725665077*	3.041	2017-2018
4A	725,665,094	*G_Chr4A_725665094*	3.041	2017-2018
4A	725,665,136	*G_Chr4A_725665136*	3.041	2017-2018
4B	641,557,790	*G_Chr4B_641557790*	3.195	2017-2018
5A	36,728,427	*G_Chr5A_36728427*	3.647	2016-2017
			6.226	2017-2018
5A	36,728,445	*G_Chr5A_36728445*	4.199	2017-2018
5A	37,883,975	*R_Chr5A_37883975*	4.926	2016-2017
			8.936	2017-2018
5A	96,804,576	*G_Chr5A_96804576*	3.269	2016-2017
			4.536	2017-2018
5A	128,847,069	*G_Chr5A_128847069*	4.247	2016-2017
			6.878	2017-2018
5A	133,359,144	*R_Chr5A_133359144*	3.661	2017-2018
5A	550,033,542	*G_Chr5A_550033542*	3.158	2017-2018
5B	485,475,432	*R_Chr5B_485475432*	3.838	2016-2017
			4.217	2017-2018
6A	162,625,801	*R_Chr6A_162625801*	3.131	2017-2018
6A	174,924,572	*R_Chr6A_174924572*	4.001	2017-2018
6A	232,386,845	*G_Chr6A_232386845*	3.131	2017-2018
6A	358,132,899	*R_Chr6A_358132899*	3.443	2017-2018
6B	21,154,867	*G_Chr6B_21154867*	3.057	2017-2018
6B	54,683,176	*G_Chr6B_54683176*	3.234	2017-2018
6B	55,007,111	*G_Chr6B_55007111*	3.234	2017-2018

6B	141,397,191	*R_Chr6B_141397191*	3.860	2017-2018
6B	163,909,622	*G_Chr6B_163909622*	3.498	2016-2017
6B	196,264,617	*G_Chr6B_196264617*	3.197	2016-2017
6B	197,163,092	*G_Chr6B_197163092*	4.990	2016-2017
6B	197,734,625	*R_Chr6B_197734625*	4.350	2016-2017
6B	198,126,267	*R_Chr6B_198126267*	4.350	2016-2017
6B	198,739,148	*R_Chr6B_198739148*	4.656	2016-2017
6B	199,742,815	*G_Chr6B_199742815*	3.449	2016-2017
6B	200,064,777	*R_Chr6B_200064777*	3.449	2016-2017
6B	201,075,162	*R_Chr6B_201075162*	4.204	2016-2017
6B	201,075,235	*R_Chr6B_201075235*	4.204	2016-2017
6B	201,075,240	*R_Chr6B_201075240*	4.204	2016-2017
6B	201,129,247	*R_Chr6B_201129247*	3.846	2016-2017
6B	201,129,392	*R_Chr6B_201129392*	4.656	2016-2017
6B	201,129,463	*R_Chr6B_201129463*	3.846	2016-2017
6B	201,315,880	*tarc2311*	3.106	2016-2017
6B	203,703,616	*G_Chr6B_203703616*	3.983	2016-2017
6B	204,388,184	*R_Chr6B_204388184*	3.465<br>3.289	2016-2017<br>2017-2018
6B	204,912,051	*G_Chr6B_204912051*	3.151	2016-2017
6B	208,793,517	*R_Chr6B_208793517*	3.465<br>3.289	2016-2017<br>2017-2018
6B	297,862,708	*G_Chr6B_297862708*	3.646	2017-2018
6B	327,409,706	*R_Chr6B_327409706*	3.646	2017-2018
6B	328,142,779	*G_Chr6B_328142779*	3.646	2017-2018
6B	431,048,550	*G_Chr6B_431048550*	3.646	2017-2018
6B	459,720,082	*G_Chr6B_459720082*	3.912	2016-2017
6B	459,720,091	*G_Chr6B_459720091*	3.912	2016-2017
6B	462,788,719	*G_Chr6B_462788719*	3.007	2016-2017
6B	462,799,706	*R_Chr6B_462799706*	3.499	2016-2017
6B	463,269,238	*G_Chr6B_463269238*	3.007	2016-2017
6B	463,305,466	*G_Chr6B_463305466*	3.007	2016-2017
6B	463,638,958	*R_Chr6B_463638958*	3.499	2016-2017
6B	470,381,538	*R_Chr6B_470381538*	4.675	2016-2017
6B	474,283,184	*R_Chr6B_474283184*	3.005	2016-2017
6B	474,283,190	*R_Chr6B_474283190*	3.005	2016-2017
6B	474,283,207	*R_Chr6B_474283207*	3.005	2016-2017
6B	474,283,263	*R_Chr6B_474283263*	3.005	2016-2017
6B	474,418,103	*R_Chr6B_474418103*	3.757	2016-2017
6B	479,120,020	*R_Chr6B_479120020*	3.788	2016-2017
6B	479,120,151	*R_Chr6B_479120151*	3.788	2016-2017

6B	483,198,040	*G_Chr6B_483198040*	3.371	2016-2017
6B	495,929,323	*G_Chr6B_495929323*	3.479	2016-2017
6B	524,731,600	*G_Chr6B_524731600*	5.812	2016-2017
6B	524,732,689	*G_Chr6B_524732689*	5.812	2016-2017
6B	525,583,750	*G_Chr6B_525583750*	5.812	2016-2017
6B	525,648,961	*G_Chr6B_525648961*	5.812	2016-2017
6B	526,203,243	*G_Chr6B_526203243*	5.105	2016-2017
6B	526,203,290	*G_Chr6B_526203290*	5.105	2016-2017
6B	526,763,360	*G_Chr6B_526763360*	3.412	2016-2017
6B	527,570,261	*G_Chr6B_527570261*	4.331	2016-2017
6B	527,570,277	*G_Chr6B_527570277*	3.539	2016-2017
6B	527,570,303	*G_Chr6B_527570303*	4.331	2016-2017
6B	665,391,529	*G_Chr6B_665391529*	3.066	2016-2017
			3.715	2017-2018
6B	665,391,536	*G_Chr6B_665391536*	3.066	2016-2017
			3.715	2017-2018
7A	4,120,994	*G_Chr7A_4120994*	3.558	2016-2017
7A	4,131,004	*G_Chr7A_4131004*	3.558	2016-2017
7A	4,371,928	*G_Chr7A_4371928*	3.558	2016-2017
7A	4,371,946	*G_Chr7A_4371946*	3.558	2016-2017
7A	4,371,964	*G_Chr7A_4371964*	3.558	2016-2017
7A	4,371,984	*G_Chr7A_4371984*	3.558	2016-2017
7A	4,422,968	*G_Chr7A_4422968*	3.558	2016-2017
7A	4,423,001	*G_Chr7A_4423001*	3.558	2016-2017
7A	4,550,469	*G_Chr7A_4550469*	3.558	2016-2017
7A	4,743,487	*G_Chr7A_4743487*	3.558	2016-2017
7A	4,743,535	*G_Chr7A_4743535*	3.558	2016-2017
7A	4,744,972	*G_Chr7A_4744972*	3.558	2016-2017
7A	4,744,978	*G_Chr7A_4744978*	3.558	2016-2017
7A	4,801,192	*G_Chr7A_4801192*	3.558	2016-2017
7A	4,868,174	*G_Chr7A_4868174*	3.558	2016-2017
7A	4,868,190	*G_Chr7A_4868190*	3.558	2016-2017
7A	6,642,404	*G_Chr7A_6642404*	3.558	2016-2017
7A	43,864,855	*G_Chr7A_43864855*	3.609	2016-2017
7A	44,382,859	*tarc0775*	3.880	2017-2018
7A	44,389,117	*G_Chr7A_44389117*	3.880	2017-2018
7A	44,389,134	*G_Chr7A_44389134*	4.584	2017-2018
7A	44,406,600	*G_Chr7A_44406600*	3.662	2016-2017
7A	121,839,265	*G_Chr7A_121839265*	3.272	2017-2018
7A	140,368,613	*G_Chr7A_140368613*	3.073	2017-2018
7A	172,559,137	*G_Chr7A_172559137*	6.041	2017-2018

7A	172,878,604	*R_Chr7A_172878604*	4.083	2017-2018
7A	172,878,864	*R_Chr7A_172878864*	4.083	2017-2018
7A	173,288,218	*G_Chr7A_173288218*	6.041	2017-2018
7A	174,421,918	*G_Chr7A_174421918*	3.644	2016-2017
7A	174,591,482	*snp305_1*	3.644	2016-2017
7A	174,951,999	*R_Chr7A_174951999*	3.644	2016-2017
7A	175,599,804	*R_Chr7A_175599804*	3.644	2016-2017
7A	176,716,132	*G_Chr7A_176716132*	3.644	2016-2017
7A	177,991,292	*G_Chr7A_177991292*	3.644	2016-2017
7A	614,394,284	*G_Chr7A_614394284*	3.215	2017-2018
7A	678,592,309	*G_Chr7A_678592309*	3.074	2017-2018
7A	714,113,522	*G_Chr7A_714113522*	3.132	2016-2017
7A	714,210,775	*G_Chr7A_714210775*	3.132	2016-2017
7B	114,697,005	*G_Chr7B_114697005*	3.012	2017-2018
7B	115,937,657	*G_Chr7B_115937657*	3.012	2017-2018
7B	612,240,988	*G_Chr7B_612240988*	3.265	2016-2017
7B	646,337,902	*G_Chr7B_646337902*	3.088	2017-2018
7B	691,199,872	*G_Chr7B_691199872*	3.751	2016-2017
7D	6,703,320	*R_Chr7D_6703320*	3.521	2016-2017
7D	7,070,623	*G_Chr7D_7070623*	3.521	2016-2017
7D	7,070,648	*G_Chr7D_7070648*	3.521	2016-2017
7D	7,096,706	*G_Chr7D_7096706*	3.521	2016-2017
7D	7,778,931	*G_Chr7D_7778931*	3.521	2016-2017
7D	7,852,608	*G_Chr7D_7852608*	3.521	2016-2017
7D	416,203,104	*G_Chr7D_416203104*	4.224	2017-2018
7D	502,841,852	*snp2273*	3.902	2017-2018
7D	503,215,094	*R_Chr7D_503215094*	3.451	2017-2018
7D	533,206,507	*G_Chr7D_533206507*	3.600	2016-2017

Supplemental Table 8 Response patterns of JWC accessions to WYMV pathotypes by combination of resistance haplotypes

Genotype of resistance loci					Number of accessions	Resistant to pathotypes I and III (%)	Resistant to pathotype I susceptible to pathotype III (%)	Susceptible to pathotypes I and III (%)	Non-infection with pathotype II (%)
2DL	3BS	5AL	6DS	7AS					
R	R	S	U	R	2	2 (16.7)	0	0	2 (5.1)
R	S	S	R	R	2	1 (8.3)	1 (4.5)	0	1 (2.6)
R	S	S	S	R	1	1 (8.3)	0	0	1 (2.6)
S	R	R	R	S	1	0	1 (4.5)	0	1 (2.6)
S	R	R	S	R	2	1 (8.3)	1 (4.5)	0	0
S	R	R	U	R	6	1 (8.3)	5 (22.7)	0	0
S	R	R	U	S	12	0	4 (18.2)	8 (12.9)	10 (25.6)
S	R	S	R	R	4	2 (16.7)	0	2 (3.2)	2 (5.1)
S	R	S	R	S	15	0	0	15 (24.2)	10 (25.6)
S	R	S	S	R	1	0	0	1 (1.6)	0
S	R	S	U	R	2	0	0	2 (3.2)	0
S	R	S	U	S	22	1 (8.3)	1 (4.5)	20 (32.3)	7 (17.9)
S	S	R	S	R	8	2 (16.7)	6 (27.3)	0	0
S	S	R	U	R	2	0	2 (9.1)	0	0
S	S	R	U	S	2	0	1 (4.5)	1 (1.6)	1 (2.6)
S	S	S	R	R	1	0	0	1 (1.6)	0
S	S	S	R	S	3	0	0	3 (4.8)	2 (5.1)
S	S	S	S	R	2	0	0	2 (3.2)	0
S	S	S	U	R	3	1 (8.3)	0	2 (3.2)	0
S	S	S	U	S	5	0	0	5 (8.1)	2 (5.1)
Total					96	12	22	62	39

Supplemental Table 9 Effect of each resistance haplotype on response to pathotypes II

Resistance haplotype	Number of accessions	Resistant	Susceptible	Not determined	PRV [a] (%)
2DL	5	4	0	1	80
3BS	67	32	22	13	47.8
5AL	33	12	15	6	36.4
6DS	26	16	5	5	61.5
7AS	36	6	22	8	16.7

a: Percentage of varieties resistant to WYMV
